# Supplementary material for: Preference and retention of daily and event-driven pre-exposure prophylaxis for HIV prevention: a prospective cohort in Can Tho city, Viet Nam
Source: BMJ Open. 2024 Feb 29;14(2):e075976. doi: 10.1136/bmjopen-2023-075976 (PMC10910397; doi:10.1136/bmjopen-2023-075976)
Supplement: Supplementary data [file bmjopen-2023-075976supp001.pdf]

**Supplementary table 1. Baseline characteristic of participants using daily PrEP by the retention** (excluding participants who decide to discontinue PrEP due to decreased HIV risk perception)

| | All participants (n=491) | Retained in care (n=232) | Lost to follow up (n=259) | P value |
|---|---|---|---|---|
| **Sex assigned at birth** | | | | |
| Male | 465 (94.7%) | 220 (94.8%) | 245 (94.6%) | 0.908 |
| Female | 26 (5.3%) | 12 (5.2%) | 14 (5.4%) | |
| **Sexual partners** | | | | |
| No answer | 1 (0.2%) | 1 (0.4%) | 0 (0.0%) | 0.295 |
| Men exclusively | 419 (85.3%) | 202 (87.1%) | 217 (83.8%) | |
| Men and women | 71 (14.5%) | 29 (12.5%) | 42 (16.2%) | |
| **HIV exposure within the past 3 days** | | | | |
| No HIV exposure | 488 (99.4%) | 231 (99.6%) | 257 (99.2%) | 0.637 |
| HIV exposure | 2 (0.4%) | 1 (0.4%) | 1 (0.4%) | |
| No answer | 1 (0.2%) | 0 (0.0%) | 1 (0.4%) | |
| **Frequency of sexual activity** | | | | |
| ≤ 2 times per week | 169 (34.4%) | 107 (46.1%) | 62 (23.9%) | <0.001 |
| >2 times per week | 264 (53.8%) | 105 (45.3%) | 159 (61.4%) | |
| No answer | 58 (11.8%) | 20 (8.6%) | 38 (14.7%) | |
| **Having sex without condom with people who were at risk of HIV within the past 6 months** | | | | |
| No | 130 (26.5%) | 53 (22.8%) | 77 (29.7%) | 0.060 |
| Yes | 312 (63.5%) | 160 (69.0%) | 152 (58.7%) | |
| No answer | 49 (10.0%) | 19 (8.2%) | 30 (11.6%) | |
| **Number of sexual partner within the past 6 months** | | | | |
| 1 sexual partner | 115 (23.4%) | 64 (27.6%) | 51 (19.7%) | 0.102 |
| At least 2 sexual partners | 370 (75.4%) | 166 (71.6%) | 204 (78.8%) | |
| No answer | 6 (1.2%) | 2 (0.9%) | 4 (1.5%) | |
| **Having sex for money or gifts within the past 6 months** | | | | |
| No | 461 (93.9%) | 225 (97.0%) | 236 (91.1%) | 0.017 |
| Yes | 21 (4.3%) | 6 (2.6%) | 15 (5.8%) | |
| No answer | 9 (1.8%) | 1 (0.4%) | 8 (3.1%) | |
| **Diagnosis and/or treatment with an STI within the past 6 months** | | | | |
| No | 439 (89.4%) | 208 (89.7%) | 231 (89.2%) | 0.006 |
| Yes | 39 (7.9%) | 23 (9.9%) | 16 (6.2%) | |
| No answer | 13 (2.6%) | 1 (0.4%) | 12 (4.6%) | |
| **Sharing needles with other people within the past 6 months** | | | | |
| No | 486 (99.8%) | 230 (100.0%) | 256 (99.6%) | 0.344 |

| | | | | |
|---|---|---|---|---|
| Yes | 0 (0.0%) | 0 (0.0%) | 0 (0.0%) | |
| No answer | 1 (0.2%) | 0 (0.0%) | 1 (0.4%) | |
| **Used PrEP within the past 6 months** | | | | |
| No | 451 (92.6%) | 199 (86.5%) | 252 (98.1%) | <0.0001 |
| Yes | 33 (6.8%) | 31 (13.5%) | 2 (0.8%) | |
| No answer | 3 (0.6%) | 0 (0.0%) | 3 (1.2%) | |
| **Willingness to pay for PrEP** | | | | |
| < $4.25/month | 100 (20.4%) | 44 (19.0%) | 56 (21.6%) | 0.101 |
| $4.25-$14.89/month | 302 (61.5%) | 138 (59.5%) | 164 (63.3%) | |
| $14.89-42.55$/month | 67 (13.6%) | 41 (17.7%) | 26 (10.0%) | |
| >$42.55/month | 1 (0.2%) | 1 (0.4%) | 0 (0.0%) | |
| Don't want/unable to pay for PrEP | 21 (4.3%) | 8 (3.4%) | 13 (5.0%) | |

**Supplementary table 3. Concerns about potential barriers to access PrEP at baseline**

| | All | Daily PrEP | ED-PrEP |
|---|---|---|---|
| Anticipated barrier to PrEP | 338 (36.5%) | 265 (39.4%) | 73 (28.9%) |
| Concern about side effects | 9 (1.0%) | 9 (1.3%) | 0 (0.0%) |
| Difficult in managing the time for visiting clinic and drug pick up PrEP | 8 (0.9%) | 5 (0.7%) | 3 (1.2%) |
| Concerned about stigma/community perception | 8 (0.9%) | 8 (1.2%) | 0 (0.0%) |
| Concerned about family finding out | 6 (0.6%) | 6 (0.9%) | 0 (0.0%) |
| Concern about cost | 6 (0.6%) | 6 (0.9%) | 0 (0.0%) |
| Concerned about stigma/community perception | 4 (0.4%) | 4 (0.6%) | 0 (0.0%) |
| Difficulty in planning sex in advance | 3 (0.3%) | 1 (0.1%) | 2 (0.8%) |
| Concerned about carrying the pills | 2 (0.2%) | 2 (0.3%) | 0 (0.0%) |
| Difficulty in taking the pills 2 hours before sex | 1 (0.1%) | 1 (0.1%) | 0 (0.0%) |
| Difficulty in remembering to take tablet everyday | 1 (0.1%) | 0 (0.0%) | 1 (0.4%) |
| Drug storage | 1 (0.1%) | 1 (0.1%) | 0 (0.0%) |
| Difficulty in managing the time for visiting clinic and drug pick up | 1 (0.1%) | 0 (0.0%) | 1 (0.4%) |
| Concerned on the comorbidity of asthma | 1 (0.1%) | 0 (0.0%) | 1 (0.4%) |
| Difficulty in transportation of PrEP | 0 (0.0%) | 0 (0.0%) | 0 (0.0%) |
| Concerned about partner finding out | 0 (0.0%) | 0 (0.0%) | 0 (0.0%) |
| Difficulty in remembering to take the following doses | 0 (0.0%) | 0 (0.0%) | 0 (0.0%) |
| Difficulty in accessing STI, hepatitis testing and/or treatment | 0 (0.0%) | 0 (0.0%) | 0 (0.0%) |

**Supplementary table 3. Challenges reported by daily PrEP users at 3, 6 and 12 months interviews**

| | 3 months N= 373 | 6 months N=235 | 12 months N=44 |
|---|---|---|---|
| **Willing to pay for PrEP per month** | | | |

| | | | |
|---|---|---|---|
| Don’t want/unable to pay for PrEP | 19 (5.1%) | 9 (3.8%) | 0 (0.0%) |
| >$42.55 | 1 (0.3%) | 1 (0.4%) | 0 (0.0%) |
| $14.89-42.55$ | 45 (12.1%) | 36 (15.3%) | 21 (47.7%) |
| $4.25-$14.89 | 240 (64.3%) | 152 (64.7%) | 22 (50.0%) |
| < $4.25 | 68 (18.2%) | 36 (15.3%) | 1 (2.3%) |
| **In the past 3 months, do you use daily PrEP or ED PrEP?** | | | |
| Both daily and ED PrEP | 17 (4.6%) | 3 (1.3%) | 0 (0.0%) |
| Daily PrEP | 296 (79.4%) | 190 (80.9%) | 36 (81.8%) |
| Any difficulties in taking PrEP medication in the past 3 months | 110 (29.5%) | 22 (9.4%) | 13 (29.5%) |
| Forgot to take one or more PrEP doses | 37 (9.9%) | 9 (3.8%) | 5 (11.4%) |
| Fitting PrEP into daily routine (for daily PrEP users) | 7 (1.9%) | 1 (0.4%) | 1 (2.3%) |
| Unsure when to take PrEP medications | 1 (0.3%) | 1 (0.4%) | 0 (0.0%) |
| Didn’t know what to do about a missed dose | 5 (1.3%) | 3 (1.3%) | 2 (4.5%) |
| Concerns about interactions with other medications | 11 (2.9%) | 5 (2.1%) | 7 (15.9%) |
| Concerns about interactions with alcohol | 2 (0.5%) | 3 (1.3%) | 2 (4.5%) |
| Concerns about interactions with hormonal therapy | 1 (0.3%) | 1 (0.4%) | 0 (0.0%) |
| Have you had any difficulties accessing PrEP services? | 131 (35.1%) | 53 (22.6%) | 18 (40.9%) |
| Difficult in managing the time for visiting clinic and drug pick up | 37 (9.9%) | 18 (7.7%) | 6 (13.6%) |
| Transportation difficulty | 11 (2.9%) | 13 (5.5%) | 13 (29.5%) |
| Concerned about partner finding out | 5 (1.3%) | 5 (2.1%) | 1 (2.3%) |
| Concerned about family finding out | 32 (8.6%) | 12 (5.1%) | 2 (4.5%) |
| Concerned about stigma/community perception | 9 (2.4%) | 5 (2.1%) | 0 (0.0%) |
| Difficulty in remembering to take tablet everyday | 17 (4.6%) | 2 (0.9%) | 0 (0.0%) |
| Carrying the pills with me | 13 (3.5%) | 7 (3.0%) | 5 (11.4%) |
| Concern about side effects | 52 (13.9%) | 12 (5.1%) | 4 (9.1%) |
| Concern about cost | 9 (2.4%) | 1 (0.4%) | 0 (0.0%) |
| Difficulty in accessing STI, Hepatitis testing and/or treatment | 5 (1.3%) | 3 (1.3%) | 1 (2.3%) |
| Health services closed due to Covid-19 | 33 (8.8%) | 21 (8.9%) | 16 (36.4%) |

| | | | |
|---|---|---|---|
| Not able to leave home due to Covid-19 lockdown | 34 (9.1%) | 27 (11.5%) | 15 (34.1%) |
| **Side effects** | | | |
| Sleepy | 1 (0.3%) | 0 (0.0%) | 0 (0.0%) |
| Nausea | 12 (3.2%) | 1 (0.4%) | 1 (2.3%) |
| Muscle pain | 1 (0.3%) | 0 (0.0%) | 0 (0.0%) |
| Loss of appetite | 2 (0.5%) | 0 (0.0%) | 0 (0.0%) |
| Headache | 3 (0.8%) | 3 (1.3%) | 2 (4.5%) |
| Dry lips | 5 (1.3%) | 2 (0.9%) | 1 (2.3%) |
| Burning | 5 (1.3%) | 2 (0.9%) | 4 (9.1%) |
| Acne | 0 (0.0%) | 0 (0.0%) | 2 (4.5%) |
| Hyperpigmentation | 0 (0.0%) | 0 (0.0%) | 0 (0.0%) |
| Fatigue | 7 (1.9%) | 6 (2.5%) | 1 (2.3%) |
| Dizziness | 7 (1.9%) | 0 (0.0%) | 0 (0.0%) |
| Dry skin | 0 (0.0%) | 0 (0.0%) | 1 (2.3%) |

**Supplementary table 4. Number of participants switching between daily and ED-PrEP during the following up period**

| | Regimen | Participants preferred a daily PrEP regimen | |
|---|---|---|---|
| **During the first 3 months of PrEP use** | Daily PrEP | 296 | 80.0% |
| | ED-PrEP | 57 | 15.4% |
| | Switching between daily and ED-PrEP | 17 | 4.6% |
| | Subtotal | 370 | 100.0% |
| **Between 3 months and 6 months of PrEP use** | Daily PrEP | 190 | 81.2% |
| | ED-PrEP | 41 | 17.5% |

| | | | |
|---|---|---|---|
| | Switching between daily and ED-PrEP | 3 | 1.3% |
| | Subtotal | 234 | 100.0% |
| **Between 9 and 12 months of PrEP use** | Daily PrEP | 36 | 83.7% |
| | ED-PrEP | 7 | 16.3% |
| | Switching between daily and ED-PrEP | 0 | 0.0% |
| | Subtotal | 43 | 100.0% |
