# Supplementary material for: Preference and retention of daily and event-driven pre-exposure prophylaxis for HIV prevention: a prospective cohort in Can Tho city, Viet Nam
Source: BMJ Open. 2024 Feb 29;14(2):e075976. doi: 10.1136/bmjopen-2023-075976 (PMC10910397; doi:10.1136/bmjopen-2023-075976)
Supplement: Author's manuscript [file bmjopen-2023-075976.draft_revisions.pdf]

BMJ Open

BMJ Open is committed to open peer review. As part of this commitment we make the peer review history of every article we publish publicly available.

When an article is published we post the peer reviewers' comments and the authors' responses online. We also post the versions of the paper that were used during peer review. These are the versions that the peer review comments apply to.

The versions of the paper that follow are the versions that were submitted during the peer review process. They are not the versions of record or the final published versions. They should not be cited or distributed as the published version of this manuscript.

BMJ Open is an open access journal and the full, final, typeset and author-corrected version of record of the manuscript is available on our site with no access controls, subscription charges or pay-per-view fees (http://bmjopen.bmj.com).

If you have any questions on BMJ Open's open peer review process please email info.bmjopen@bmj.com

BMJ Open

Preference and retention of daily and event-driven pre-exposure prophylaxis: a prospective cohort in Can Tho city, Viet Nam

Journal:	*BMJ Open*
Manuscript ID	bmjopen-2023-075976
Article Type:	Original research
Date Submitted by the Author:	26-May-2023
Complete List of Authors:	Nguyen, Van Thi Thuy; World Health Organization, WHO Viet Nam<br>Dat, Vu Quoc; Hanoi Medical University, Department of Infectious Diseases; Hanoi Medical University, Hanoi Medical University Hospital<br>Truc, Huynh Minh; Can Tho city Center for Disease Control<br>Nguyen Anh Thu, Pham; Can Tho city Center for Disease Control<br>Linh, Doan Thi Thuy; Vietnam Administration for HIV/AIDS Control<br>Johnson, Cheryl; World Health Organization, Department of HIV/AIDS<br>Baggaley, R; World Health Organization<br>Phan, Huong; Vietnam Administration of HIV/AIDS control
Keywords:	HIV & AIDS < INFECTIOUS DISEASES, Health Services Accessibility, PREVENTIVE MEDICINE

SCHOLARONE™
Manuscripts

BMJ

I, the Submitting Author has the right to grant and does grant on behalf of all authors of the Work (as defined in the below author licence), an exclusive licence and/or a non-exclusive licence for contributions from authors who are: i) UK Crown employees; ii) where BMJ has agreed a CC-BY licence shall apply, and/or iii) in accordance with the terms applicable for US Federal Government officers or employees acting as part of their official duties; on a worldwide, perpetual, irrevocable, royalty-free basis to BMJ Publishing Group Ltd ("BMJ") its licensees and where the relevant Journal is co-owned by BMJ to the co-owners of the Journal, to publish the Work in this journal and any other BMJ products and to exploit all rights, as set out in our licence.

The Submitting Author accepts and understands that any supply made under these terms is made by BMJ to the Submitting Author unless you are acting as an employee on behalf of your employer or a postgraduate student of an affiliated institution which is paying any applicable article publishing charge ("APC") for Open Access articles. Where the Submitting Author wishes to make the Work available on an Open Access basis (and intends to pay the relevant APC), the terms of reuse of such Open Access shall be governed by a Creative Commons licence – details of these licences and which Creative Commons licence will apply to this Work are set out in our licence referred to above.

Other than as permitted in any relevant BMJ Author's Self Archiving Policies, I confirm this Work has not been accepted for publication elsewhere, is not being considered for publication elsewhere and does not duplicate material already published. I confirm all authors consent to publication of this Work and authorise the granting of this licence.

For peer review only

Preference and retention of daily and event-driven pre-exposure prophylaxis: a prospective cohort in Can Tho city, Viet Nam

Running Title: Pre-exposure prophylaxis for HIV in Vietnam

Van Thi Thuy Nguyen[1¥], Vu Quoc Dat[2,3¥*], Huynh Minh Truc[4], Pham Nguyen Anh Thu[4], Doan Thi Thuy Linh[5], Cheryl Johnson[6], Rachel Baggaley[6], Phan Thi Thu Huong[5]

[1] World Health Organization - Vietnam Country Office, Hanoi, Vietnam

[2] Department of Infectious Diseases, Hanoi Medical University, Hanoi, Vietnam

[3] Hanoi Medical University Hospital, Hanoi Medical University, Hanoi, Vietnam

[4] Can Tho city Center for Disease Control and Prevention

[5] Vietnam Administration for HIV/AIDS Control, Vietnam

[6] World Health Organization, Global HIV, Viral hepatitis and STIs programmes, Geneva, Switzerland

[¥] Co-first author

*Corresponding author: Vu Quoc Dat, MD, PhD. Email: datvq@hmu.edu.vn. Postal address: No 1 Ton That Tung Street, Hanoi, Viet Nam.

For peer review only

Abstract

Objective

Pre-exposure prophylaxis (PrEP) was introduced in Vietnam in 2017, but data on PrEP preference and retention beyond 3 months are limited. We aimed to evaluate preferences for PrEP, factors influencing PrEP choice, barriers to PrEP access and retention.

Methods

This is a prospective cohort study in Can Tho, Vietnam. Participants who were eligible for PrEP and provided informed consent were interviewed at baseline on demographic information, willingness to pay, reasons for choosing their PrEP regimen and the anticipated difficulties in taking PrEP and followed-up at 3 months, 6 months and 12 months after PrEP initiation.

Findings

Between May 2020 and April 2021, 926 individuals at substantial risk for HIV were enrolled for PrEP. Of whom 673 (72.7%) choose daily PrEP and 253 (27.3%) choose ED-PrEP. Majority of participants were men (92.7%) and only 6.8% were women and 0.5% were female transgenders. Median age was 24 years (IQR 20-28) and 84.7% participants reported as exclusively homosexual. The three most common reasons for choosing daily PrEP were PrEP effectiveness (24.3%), unplanning for sex (22.9%), and for choosing ED-PrEP were PrEP effectiveness (22.7%), convenient (18.0%) and easier adherence 12.0%). Only 7.8% of PrEP users indicated their unwillingness to pay for PrEP and 76.4% would be willing to pay if PrEP were less than $15 per month. The proportion of retention at 12 months was 43.1% and 99.2% in daily PrEP and ED-PrEP users, respectively.

Conclusions

Event-driven PrEP was preferred by more than a quarter of 23.5% of the participants and there was a little concern about adverse events. High retention rate was reported by ED-PrEP users. Future research to inform implementation of PrEP in Vietnam is needed to develop ways of measuring adherence to ED-PrEP more accurately and to understand and address difficulties in taking daily PrEP use.

Keywords: PrEP, pre-exposure prophylaxis, daily, event-driven, on-demand PrEP, MSM, retention, Vietnam

Strengths and limitations of this study

- We conducted the first study on preference, retention and factors associated with these in PrEP use in Vietnam.

For peer review only - http://bmjopen.bmj.com/site/about/guidelines.xhtml

- We found that event driven PrEP was preferred over daily PrEP and was associated with high retention rate at 12 months.
- The major limitations related to the study design of single center

For peer review only

For peer review only - http://bmjopen.bmj.com/site/about/guidelines.xhtml

Introduction

As of December 2020, Vietnam reported that there were 215,220 people with HIV, in which there were 12,200 new HIV infections and 1,681 deaths in 2020 [1]. The HIV epidemic in Viet Nam is concentrated in key populations including people who inject drugs (PWID), men who have sex with men (MSM) and female sex workers (FSWs). It is estimated that there are approximately 200,000 MSM in Vietnam[2]. In recent years, HIV prevalence has increased in the MSM population, from 5.1% in 2015 to 13.3% in 2020, while prevalence was stable in PWID populations (12.7% in 2019) and FSWs (3.1% in 2020) [1].

Since 2016, the World Health Organization (WHO) has recommended oral pre-exposure prophylaxis (PrEP) as a way to further reduce new infections where HIV incidence and HIV risk is high. Following this guidance, between June and December 2017, Vietnam updated national guidelines to include oral PrEP and started initial PrEP implementation in two cities: Hanoi and Ho Chi Minh city. PrEP implementation in Vietnam has continued to expand through this programme and as of August of 2021, there were nearly 32,000 persons using PrEP in 200 PrEP clinics in 28 out of 63 provinces throughout the country[3]. And the current national guidelines recommend daily-PrEP (tenofovir disoproxil fumarate (TDF) co-formulated with emtricitabine (FTC) or lamivudine (3TC)) for populations at substantial risk and event-driven PrEP (ED-PrEP) (TDF/XTC) for MSM who have less frequent sex (less than twice a week) [4].

While oral PrEP continues to expand and be an effective option for many, recent evidence has highlighted that more differentiated service delivery options are needed. In particular, event-driven (ED)-PrEP which removes the need for daily doses and is used prior to and after high risk sex has been shown to reduce HIV transmission by up to 86% among MSM[5]. Studies have also shown that MSM may often prefer ED-PrEP over daily oral PrEP because of its convenience and flexibility. In a survey in the USA, 74.3% of MSM who were hesitant to start daily PrEP indicated that they would be more willing to try ED-PrEP [6]. In Thailand some PrEP users considered daily regimens the easiest to use, as it could be incorporated into daily routines and did not require planning for sex. These men expressed concerns, however, about the long-term safety and affordability of daily oral dosing [7]. Study participants appreciated ED-PrEP for minimizing drug exposure and potential adverse events. They considered ED-PrEP an attractive choice for MSM who had infrequent sex, were able to plan for sex, and had the ability to take the post-sex dose [7].

Despite the potential benefits of ED-PrEP, it is little known about preference and uptake of ED-PrEP r among MSM in Vietnam. Thus, this study aims to assess both preferences as well as actual

For peer review only - http://bmjopen.bmj.com/site/about/guidelines.xhtml

uptake and continuation of daily and ED-PrEP among MSM in Vietnam to inform programming and planning. In addition, difficulties related to PrEP uptake and continuation including COVID-19-related issues were explored to inform future differentiated PrEP service delivery models.

Methods

Study design and participants

We conducted a prospective study in all 11 PrEP clinics in Can Tho which is considered as a capital province of the Mekong river delta with large MSM population with highest HIV prevalence in this key population (22.7%)[2]. MSM were referred to the PrEP clinics from community-based HIV testing led by MSM groups or self-referral. PrEP eligibility was evaluated following the national guideline: (1) confirmed HIV-negative status, (2) no signs and symptoms of acute HIV infection and (3) at substantial risk for HIV infection within past 6 months. We defined substantial risk as any of the following: individual engaged in condomless anal or vaginal sex, having at least 2 sexual partners, reported sexual partner with substantial risk for HIV infection, or having a sexual partner living HIV but not on ART or with unknown/detectable viral load (>200 copies/ml), previously diagnosed with a sexually transmitted disease (STD), and having multiple courses of PEP and continued sexual risk behaviour. Only eligible participants aged 16 years and over who agreed to participate and provide written informed consent were recruited for the study.

Study procedure and data collection

In the community-based setting, screening for PrEP is part of HIV post-test counselling (HTC) including counselling on the options of daily and ED-PrEP [8]. Clients who were interested in PrEP will be referred to PrEP clinic. At the PrEP clinics, clients were screened by a standard form to evaluate their behavioural risk to assess eligibility. ED-PrEP were offered for MSM who have infrequent sex (≤ 2 times per week on average) and are usually able to plan for sex at least two hours in advance, or who can delay sex for at least two hours or their own preference of ED-PrEP.. During screening, the clinic staff explained what PrEP is, the benefits and differences between daily PrEP and ED-PrEP and let the client decide what their preference was. After the clients choose their preferred PrEP, they were invited to participate in the study and provide written informed consents. Daily PrEP regimens were offered based on the availability of the antiretroviral including tenofovir disoproxil fumarate/emtricitabine (TDF/FTC) or tenofovir disoproxil fumarate/lamivudine (TDF/3TC) or TDF. ED- PrEP regimens were offered as TDF/FTC or TDF/3TC

National guidelines on PrEP implementation requested all PrEP clients are followed up regularly at health facilities at one and two months after PrEP initiation, and every 3 months thereafter. We used a questionnaire consisting of six questions on willingness to pay (closed-end questions) and the

For peer review only - http://bmjopen.bmj.com/site/about/guidelines.xhtml

potential barriers to use PrEP (semi-structured and open-end questions) to interview participants at enrolment, 3-month, 6-month, and 12-month visits after PrEP use. PrEP users were monitored following MOH guidelines including HIV testing and continuation. Continuation of PrEP was defined if PrEP users who come back to pick up drugs (for daily PrEP) or self-reported to adherence (for ED-PrEP) at the corresponding following up visits after initiation at 3-, 6- and 12-month visits.

Data Analysis

Participants' responses to the open-ended questions were coded by an independent investigator (VQD). Statistical analysis was performed using the IBM SPSS Statistics for Windows, Version 27.0 (Armonk, NY: IBM Corp). We used conventional descriptive statistics to summarise the characteristics of the study's participants and their views on PrEP. Retention rate was calculated by dividing the number of PrEP users retained at 3, 6 and 12 months of PrEP by the total number of clients enrolled in PrEP study and multiplying by 100. Multivariable Cox regression was used to estimate adjusted hazard ratio (aHR) and 95% CIs for PrEP retention by selected baseline characteristics. P-value < 0.05 were considered statistically significant.

Ethics

The study was approved by Hanoi Medical University Institutional Ethical Review Bboard (IRB VN01.001/IRB 0000312/FWA 00004148), and WHO Wester Pacific Regional Office Ethical Review Committee (2020.4.VTN.1.HSI).

Results

Between May 2020 and Apr 2021, we enrolled 926 clients of whom 253 (27.3%) choose ED-PrEP and 673 (72.7%) choose daily PrEP at enrolment. Participants were follow-up until 31 December 2022. Table 1 and Supplementary table 1 show the characteristics of enrolled participants. The median age was 24 years (IQR 20-28) and ranging from 16 to 51 years. There were 12 participants aged <18 years old (1.3%). The majority of men participants reported their exclusive sex with men (784/926 or 84.7%) and there was no significant difference in age and gender identity between groups of daily PrEP and ED-PrEP.

At baseline, 94.1% (871/926) participants responded to an open-ended question on the reasons that determined their PrEP preference after the consultation with the clinic staff. The response rate for reporting the reasons PrEP preference was not significant between the groups of daily PrEP (94.8%; 638/673) and ED-PrEP (233/253 or 91.1%). The most common reasons for choosing daily-PrEP was the effectiveness of PrEP in preventing HIV infection (155/638 or 24.3%), no need to plan for sex in advance (146/638 or 22.9%) and easy for adherence (121/638 or 19.0%). Among participants who

For peer review only - http://bmjopen.bmj.com/site/about/guidelines.xhtml

chose ED-PrEP, the most common reasons were the PrEP effectiveness (53/233 or 22.7%), convenience (42/233 or 18%) and easy for adherence (28/233 or 12.0%). The detailed reasons for PrEP preference at baseline was showed in the Table 2. Among 338/926 (36.5%) participants who anticipated barriers to PrEP at baseline, only 22/338 (6.5%) (17/265 preferred daily PrEP and 5/73 preferred ED-PrEP) expressed the specific concerns on PrEP (Supplementary table 2).

Regarding the willingness to pay for PrEP, most (76.4%) were willing to pay for PrEP if less than $15 per month, while only some (7.8%) said they would not pay or felt they were unable to pay for PrEP at any cost. The proportions of clients willing to pay at different prices were statistically different between daily and ED-PrEP groups (Table 1).

The median follow-up time was 284 days (IQR 102–367) among 926 participants who initiated PrEP, 214 days (IQR 60-323) in participants choosing daily PrEP and 363 days (IQR 319-389) in participants choosing ED-PrEP. By the end of the study, 261/926 (28.2%) patients were lost to follow- up, 186/926 (20.1%) discontinued and 479/926 (51.7%) were on PrEP. The majority of the loss to follow-up occurred within the first 3 months of enrolment (159/261 or 60.9%) and among those taking daily PrEP (259/261 or 99.2%). The overall retention rates at 3, 6 and 12 months in the daily PrEP group were 72.6% (439/605), 64.5% (363/563) and 43.1% (150/198), respectively with the median time of lost to follow-up of 60 days. The retention rates in the ED-PrEP group were 99.2% (251/253) at 3 and 6 months and 99.4% (158/159) at 12 months. Of 186 participants who discontinued PrEP, reasons reported for discontinuation were no longer sexually active (87/186 or 46.8%), moving to a new place (85/186 or 45.7%), diagnosed with HIV (seroconversion) (7/186 or 3.8%), participant preference, medication related toxicities and diagnosed with HBV (each of 2/185 or 1.1%) and COVID-19-related quarantine (1/186 or 0.5%).

Of participants who started daily PrEP and completed the interview, the proportions of participants reported any PrEP side effects were 32/341 (8.6%) at 3 months, 12/235 (5.1%) at 6 months and 5/44 (11.4%) at 12 months. The detail of reported side effects was listed in the Supplementary table 3.

During COVID-19 pandemic, Can Tho city was lockdown due to COVID-19 (e.g from July – October 2021). However, as shown in the Figure 2, a larger percentage of participants discontinued PrEP before the lockdown period. There were only 40/261 (15.3%) participants who lost to follow up within 4 months of lockdown and before of the study completion.

We also carried out multivariable Cox regression to determine factors associated with PrEP retention among daily PrEP participants (continued daily PrEP group vs lost-to-follow-up group). The results indicate that factors associated with daily PrEP continuation were less frequency of sex (less than

For peer review only - http://bmjopen.bmj.com/site/about/guidelines.xhtml

twice per week) (aHR 2.047, 95%CI 1.463-2.864) and no anticipation of barriers to PrEP use (aHR 1.379, 95%CI 1.004-1.893) (Table 3). Due to small number of lost to follow-up among ED-PrEP users, we were unable to execute multivariable Cox regression for this group.

Discussion

This study is the first prospective study in Viet Nam to explore the preference, retention and factors associated with PrEP continuation. We found that among individuals who were eligible for PrEP in this study, more than a quarter preferred ED-PrEP over daily PrEP. The proportion of the MSM preferred ED-PrEP in our study was similar to the studies in high income countries, including Belgium (23.5%) [9], Netherland (26.7%)[10] and Australia (20%)[11] and but was lower than countries in Asia, such as Taiwan (56%)[12] and China (57.1%)[13] and in Africa such as West Africa (74%)[14]. We reported a great variety of individual factors determine the choices for their PrEP regimens, mostly related to the participants' perceptions of PrEP efficacy in prevention of HIV transmission, safety, perceived adherence and convenience. In a qualitative study in 857 MSM on daily PrEP and 301 MSM on ED-PrEP in Netherlands, preference of PrEP was reported to include frequency of sex, expected adherence, perceived safety, efficacy and burden of the pills and anticipated side effects[15]. In a prospective study in 1000 MSM who use oral PrEP in China, the multivariable marginal effect analysis show that factors associated with an increased preference for daily versus ED-PrEP were currently being married to or living with a female (adjusted marginal effect = −0.146 [95% CI: −0.230, −0.062], p = 0.001), number of male sexual partners in the previous six months (adjusted marginal effect = 0.003, 95% CI: 0.000- 0.005], p = 0.034) and a subjective assessment of being very high risk of HIV infection (adjusted marginal effect size = 0.105 [95% CI: 0.012, 0.198], p = 0.027)[13].

For PrEP retention, we found that those on ED-PrEP had greater continuation rate compared to those opting for daily oral PrEP at 12 months (99.2% and 43.1%, p<0.001 respectively). Daily PrEP retention has been reported in different studies with large variations by study design, PrEP delivery approaches and countries. The proportion of retention at 12 months ranged from 43% in a study of 5,583 MSM from 2012 to 2017 in 6 clinical sites in United State [16], 72.3% in a cohort of 1347 PrEP users in Belgium between 2017 and 2020[17], 83% in 450 MSM in Brazil between 2014 and 2016[18] to 91.8% in a study of 400 MSM in 12 urban US cities in 2013 [19]. In a randomized control trial with 119 MSM in Hong Kong, the daily and ED PrEP retention at 32 week was 86% and 87% respectively. Proportion of retention to ED-PrEP among Thai MSM aged 15-19 years was 88.9%, 95% CI: 73.9-96.9%) at 6 months[20]. The definition of retention for PrEP was varied and made it difficult to compare the proportion of retention across the studies. It was conventionally defined as the return for follow-up every 3 months[21] or attendance at a specific timepoint (eg. 3, 6 and 12 months) with a

For peer review only - http://bmjopen.bmj.com/site/about/guidelines.xhtml

time window (±30 days) while clients may not attend a follow up visit with a precise intervals[22], especially in case of ED-PrEP. In addition, concepts of retention are changing and that people come during periods of risk and have different needs. Thus, effective use of PrEP is increasingly being used while adherence and retention are not. Our findings suggest more diverse and flexible PrEP models might lead to better use and engagement from clients. These results can also be leveraged to improve oral daily PrEP by making services and follow-up more differentiated including use of HIVST use for PrEP continuation. We found that the majority of MSM in Can Tho were willing to pay for PrEP (92.2%). However, 82.8% (707/854) participants in our study indicated that their willingness to pay was low (<15$/month) (the average income per person in Mekong delta was 3,713,000 VND[23] or approximately US160.3, at the exchange rate US$1=23,159.8 VND in 2021[24]) while 65% of respondents in Thailand willing to pay US$25 (monthly average income per person was US$478.1 in 2012[25]) and 88.9% of respondents in China would like to pay > US$14 per month for PrEP (monthly average income per person was US$ 867.3 in 2020[25]).

We noticed that the number of new registration for PrEP and the number of lost-to-follow up were highest between December 2021 and January 2022. In Viet Nam, December was designated for the HIV action month with many events promoted for HIV interventions including PrEP, which may have been related to the increase in the new registration and also high rate of lost-to-follow up thereafter.

Limitation

Our limitation in this study was the patients were recruited from a single province; thus, they were highly selected and may not represent all PrEP users in Vietnam. In addition, the self-report on challenges during PrEP taking could be recall bias.

Conclusion

Individuals at substantial risk for HIV especially MSM in Can Tho, Vietnam were motivated to choose PrEP by their beliefs about the safety, efficacy and, frequency of sex and expected adherence with little concerns about side effects and specific barriers to use PrEP. ED-PrEP was desirable and have better retention in this cohort study. The overall willingness to pay was lower than other countries in Asia. The high proportion of perceived challenges to daily PrEP impacted on the retention in care among daily PrEP users. The ED-PrEP is desirable and led to better retention; however, further research is needed to provide more insight into reasons for loss to follow up, indicators of retention in ED-PrEP use and more flexible PrEP delivery models to make it easier to users.

For peer review only - http://bmjopen.bmj.com/site/about/guidelines.xhtml

Funding

The study was funded by Viet Nam - WHO Country Office.

Contributors

VNTT, PTTH conceived and designed the study. VQD analysed the data and wrote the first draft of the report. VTTN, PTTH, HMT, PTAT, DTN, NHH and DTTL involved to the acquisition and interpretation of data. VTTN, RB, SD and PTTH reviewed the manuscript and provided substantial contribution. All authors contributed to the manuscript finalization and approved the final report.

Competing interests

We declare no competing interest.

Acknowledgments

We are very grateful to the health staff at Can Tho CDC and the participating PrEP clinics in Can Tho who contributed to the implementation of the study.

For peer review only

For peer review only - http://bmjopen.bmj.com/site/about/guidelines.xhtml

Table 1. Characteristic of participants by the initial PrEP preference

Characteristics	All participants (n=926)	Participants preferred a daily PrEP regimen (n=673)	Participants preferred ED PrEP regimen (n=253)	p value
**Gender identity**				<0.001
Male	858 (92.7%)	608 (90.3%)	250 (98.8%)	
Female	63 (6.8%)	61 (9.1%)	2 (0.8%)	
Trans female	5 (0.5%)	4 (0.6%)	1 (0.4%)	
**Age (median, IQR) (years)**	24 (20-28)	24 (20-28)	23 (21-27)	
**Sexual partners**				0.568
No answer	3 (0.3%)	3 (0.4%)	0 (0.0%)	
Men exclusively	784 (84.7%)	569 (84.5%)	215 (85.0%)	
Men and women	139 (15%)	101 (15%)	38 (15%)	
**HIV exposure within the past 3 days**				0.224
No HIV exposure	915 (98.8%)	667 (99.1%)	248 (98.0%)	
HIV exposure	10 (1.1%)	5 (0.7%)	5 (2.0%)	
No answer	1 (0.1%)	1 (0.1%)	0 (0.0%)	
**Frequency of sexual activity**				<0.001
≤ 2 times per week	279 (32.1%)	228 (37.0%)	51 (20.2%)	
>2 times per week	561 (64.6%)	366 (59.4%)	195 (77.4%)	
No answer	28 (3.2%)	22 (3.6%)	6 (2.4%)	
**Having sex without condom with people who were at risk of HIV within the past 6 months**				<0.001
No	352 (38.0%)	183 (27.2%)	169 (66.8%)	
Yes	502 (54.2%)	424 (63.0%)	78 (30.8%)	
No answer	72 (7.8%)	66 (9.8%)	6 (2.4%)	
**Number of sexual partners within the past 6 months**				<0.001
1 sexual partner	205 (22.1%)	171 (25.4%)	36 (14.2%)	
At least 2 sexual partners	703 (75.9%)	495 (73.6%)	208 (82.2%)	

For peer review only - http://bmjopen.bmj.com/site/about/guidelines.xhtml

No answer	16 (1.7%)	7 (1.0%)	9 (3.6%)	
**Having sex for money or gifts within the past 6 months**				0.002
No	835 (90.2%)	619 (92.0%)	216 (85.4%)	
Yes	60 (6.5%)	32 (4.8%)	28 (11.1%)	
No answer	31 (3.3%)	22 (3.3%)	9 (3.6%)	
**Diagnosis and/or treatment with an STI within the past 6 months**				<0.001
No	793 (85.6%)	597 (88.7%)	196 (77.5%)	
Yes	103 (11.1%)	51 (7.6%)	52 (20.6%)	
No answer	30 (3.2%)	25 (3.7%)	5 (2.0%)	
**Sharing needles with other people within the past 6 months**				0.387
No	917 (99.5%)	664 (99.3%)	253 (100.0%)	
Yes	2 (0.2%)	2 (0.3%)	0 (0.0%)	
No answer	3 (0.3%)	3 (0.4%)	0 (0.0%)	
**Used PrEP within the past 6 months**				0.001
No	874 (94.8%)	623 (93.1%)	251 (99.2%)	
Yes	44 (4.8%)	42 (6.3%)	2 (0.8%)	
No answer	4 (0.4%)	4 (0.6%)	0 (0.0%)	
**Willingness to pay for PrEP**				<0.001
< $4.25/month	196 (21.2%)	130 (19.3%)	66 (26.1%)	
$4.25-$14.89/month	511 (55.2%)	407 (60.5%)	104 (41.1%)	
$14.89-42.55$/month	145 (15.7%)	91 (13.5%)	54 (21.3%)	
>$42.55/month	2 (0.2%)	2 (0.3%)	0 (0.0%)	
Don't want/unable to pay for PrEP	72 (7.8%)	43 (6.4%)	29 (11.5%)	

Figure 1. Flowchart of study participants.

For peer review only - http://bmjopen.bmj.com/site/about/guidelines.xhtml

Table 2. Reasons for choosing PrEP at baseline

Reasons	All participants	Daily PrEP	ED-PrEP
**Number of sex partners**			
Few sex partners	25 (2.9%)	25 (3.9%)	0 (0.0%)
Multiple sex partners	72 (8.3%)	71 (11.1%)	1 (0.4%)
**Frequency of sex**			
Infrequent sex	19 (2.2%)	7 (1.1%)	12 (5.2%)
Frequent sex	104 (11.9%)	96 (15.0%)	8 (3.4%)
Effectiveness of PrEP	208 (23.9%)	155 (24.3%)	53 (22.7%)
Unplanning for sex	155 (17.8%)	146 (22.9%)	9 (3.9%)
Easy to remember or adherence	149 (17.1%)	121 (19.0%)	28 (12.0%)
Convenience	79 (9.1%)	37 (5.8%)	42 (18.0%)
Self protection from HIV	60 (6.9%)	38 (6.0%)	22 (9.4%)
No specify	60 (6.9%)	45 (7.1%)	15 (6.4%)
Safe	52 (6.0%)	37 (5.8%)	15 (6.4%)
Match the personal risk	50 (5.7%)	45 (7.1%)	5 (2.1%)
Staying with partner	33 (3.8%)	33 (5.2%)	0 (0.0%)
Having HIV positive sexual partner	29 (3.3%)	28 (4.4%)	1 (0.4%)
Wanting to try	27 (3.1%)	1 (0.2%)	26 (11.2%)
Taking fewer pills	24 (2.8%)	15 (2.4%)	9 (3.9%)
Self-esteem	20 (2.3%)	20 (3.1%)	0 (0.0%)
Having vaginal sex	18 (2.1%)	18 (2.8%)	0 (0.0%)
Don't want to use condom	17 (2.0%)	8 (1.3%)	9 (3.9%)
Free of charge	16 (1.8%)	4 (0.6%)	12 (5.2%)
Afraid of condom broken	10 (1.1%)	0 (0.0%)	10 (4.3%)
Afraid of HIV infection	7 (0.8%)	2 (0.3%)	5 (2.1%)
Want to have a child	3 (0.3%)	3 (0.5%)	0 (0.0%)
Partner takes daily PrEP	3 (0.3%)	1 (0.2%)	2 (0.9%)
HBV infection	3 (0.3%)	3 (0.5%)	0 (0.0%)
Protect the family	2 (0.2%)	2 (0.3%)	0 (0.0%)
New programme	2 (0.2%)	0 (0.0%)	2 (0.9%)
Doubt about their partners fidelity	2 (0.2%)	1 (0.2%)	1 (0.4%)
Having oral sex	1 (0.1%)	1 (0.2%)	0 (0.0%)
Frequent exposure to blood	1 (0.1%)	1 (0.2%)	0 (0.0%)
Check for the body tolerance	1 (0.1%)	1 (0.2%)	0 (0.0%)

For peer review only - http://bmjopen.bmj.com/site/about/guidelines.xhtml

Table 3. Multivariate Cox Regression predicting the retention in daily PrEP in Can Tho (*n* = 414)

Variables	Adjusted odd ratio (95%CI)	P value
**Age (1-yr. increment)**	1.012 (0.984-1.040)	0.403
**Gender identity**		
Male	1	
Female/trans female	1.812 (0.900-3.650)	0.096
**Sexual partners**		
Men and women	1	
Men exclusively	0.831 (0.538-1.283)	0.403
**Frequency of sexual activity**		
>2 times per week	1	
≤ 2 times per week	2.047 (1.463-2.864)	**0.000**
No answer	0.532 (0.246-1.150)	0.109
**Having sex without condom with people who were at risk of HIV within the past 6 months**		
No	1	
Yes	1.444 (1.028-2.031)	**0.034**
**Number of sexual partners within the past 6 months**		
≥2 partners	1	
<2 partners	0.746 (0.531-1.049)	0.092
**Having sex for money or gifts within the past 6 months**		
Yes	1	
No	1.045 (0.319-3.426)	0.942
**Diagnosis and/or treatment with an STI within the past 6 months**		
No	1	
Yes	0.692 (0.398-1.204)	0.193
**Used PrEP within the past 6 months**		
No		
Yes	0.923 (0.602-1.415)	0.713
**Willingness to pay for PrEP**		
Don't want/unable to pay for PrEP	1	
Willing to pay	1.103 (0.486-2.505)	0.814
**Anticipated barrier to PrEP (1)**		
No	1	
Yes	1.379 (1.004-1.893)	**0.047**

For peer review only - http://bmjopen.bmj.com/site/about/guidelines.xhtml

REFERENCES

1. Ministry of Health. *Report on HIV/AIDS prevention in 2020.* Hanoi 4 Feb 2021. 124/BC-BYT.
2. The Joint United Nations Programme on HIV and AIDS (UNAIDS), Vietnam Administration of AIDS Control (VAAC). *A review of HIV prevention in Vietnam 2019.* 2019.
3. Vietnam Administration of AIDS Control (VAAC). Interim guidance on implementation of PrEP in students. 2022.
4. Ministry of Health. Guidelines for the care and treatment of people living with HIV/AIDS. In: Vietnam Administration of AIDS Control (VAAC), ed. Issued with decision No. 5968/QĐ-BYT dated on 31/12/2021 ed2021.
5. Molina JM, Charreau I, Spire B, et al. Efficacy, safety, and effect on sexual behaviour of on-demand pre-exposure prophylaxis for HIV in men who have sex with men: an observational cohort study. *Lancet HIV.* 2017;4(9):e402-e410.
6. Beymer MR, Gildner JL, Holloway IW, Landovitz RJ. Acceptability of Injectable and On-Demand Pre-Exposure Prophylaxis Among an Online Sample of Young Men Who Have Sex with Men in California. *LGBT Health.* 2018;5(6):341-349.
7. Chemnasiri T, Varangrat A, Amico KR, et al. Facilitators and barriers affecting PrEP adherence among Thai men who have sex with men (MSM) in the HPTN 067/ADAPT Study. *AIDS Care.* 2020;32(2):249-254.
8. Viet Nam Authority for HIV/AIDS Control. Guidance for implemenmentation of pre-exposure prophylaxis (PrEP). Vol Letter 133/AIDS-DT dated 12.03.2020. Hanoi: Viet Nam Authority for HIV/AIDS Control,; 2020.
9. Reyniers T, Nostlinger C, Laga M, et al. Choosing Between Daily and Event-Driven Pre-exposure Prophylaxis: Results of a Belgian PrEP Demonstration Project. *J Acquir Immune Defic Syndr.* 2018;79(2):186-194.
10. Coyer L, van den Elshout MAM, Achterbergh RCA, et al. Understanding pre-exposure prophylaxis (PrEP) regimen use: Switching and discontinuing daily and event-driven PrEP among men who have sex with men. *EClinicalMedicine.* 2020;29-30:100650.
11. Vaccher SJ, Gianacas C, Templeton DJ, et al. Baseline Preferences for Daily, Event-Driven, or Periodic HIV Pre-Exposure Prophylaxis among Gay and Bisexual Men in the PRELUDE Demonstration Project. *Front Public Health.* 2017;5:341.
12. Wu HJ, Wen-Wei Ku S, Chang HH, Li CW, Ko NY, Strong C. Imperfect adherence in real life: a prevention-effective perspective on adherence to daily and event-driven HIV pre-exposure prophylaxis among men who have sex with men - a prospective cohort study in Taiwan. *Journal of the International AIDS Society.* 2021;24(5):e25733.
13. Zhang J, Xu JJ, Wang HY, et al. Preference for daily versus on-demand pre-exposure prophylaxis for HIV and correlates among men who have sex with men: the China Real-world Oral PrEP Demonstration study. *Journal of the International AIDS Society.* 2021;24(2):e25667.
14. Laurent C, Dembele Keita B, Yaya I, et al. HIV pre-exposure prophylaxis for men who have sex with men in west Africa: a multicountry demonstration study. *Lancet HIV.* 2021;8(7):e420-e428.
15. Zimmermann HM, Eekman SW, Achterbergh RC, et al. Motives for choosing, switching and stopping daily or event-driven pre-exposure prophylaxis - a qualitative analysis. *Journal of the International AIDS Society.* 2019;22(10):e25389.
16. Rusie LK, Orengo C, Burrell D, et al. Preexposure Prophylaxis Initiation and Retention in Care Over 5 Years, 2012-2017: Are Quarterly Visits Too Much? *Clin Infect Dis.* 2018;67(2):283-287.
17. Rotsaert A, Reyniers T, Jacobs BKM, et al. PrEP user profiles, dynamics of PrEP use and follow-up: a cohort analysis at a Belgian HIV centre (2017-2020). *Journal of the International AIDS Society.* 2022;25(7):e25953.

For peer review only - http://bmjopen.bmj.com/site/about/guidelines.xhtml

18. Grinsztejn B, Hoagland B, Moreira RI, et al. Retention, engagement, and adherence to pre-exposure prophylaxis for men who have sex with men and transgender women in PrEP Brasil: 48 week results of a demonstration study. *Lancet HIV.* 2018;5(3):e136-e145.
19. Hosek SG, Rudy B, Landovitz R, et al. An HIV Preexposure Prophylaxis Demonstration Project and Safety Study for Young MSM. *J Acquir Immune Defic Syndr.* 2017;74(1):21-29.
20. Traikiatphum J, Wongharn P, Moonwong J, et al. Retention in event-driven PrEP among young Thai men who have sex with men at risk of HIV acquisition. *International journal of STD & AIDS.* 2022;33(8):799-805.
21. Centers for Disease Control and Prevention. US Public Health Service: Preexposure prophylaxis for the prevention of HIV infection in the United States—2017 Update: a clinical practice guideline.: https://www.cdc.gov/hiv/pdf/risk/prep/cdc-hiv-prep-guidelines-2017.pdf.
22. Chan PA, Patel RR, Mena L, et al. Long-term retention in pre-exposure prophylaxis care among men who have sex with men and transgender women in the United States. *Journal of the International AIDS Society.* 2019;22(8):e25385.
23. General Statistics Office of Vietnam - Ministry of Planning and Investment. Announcement of population survey of life costing. 2022; https://www.gso.gov.vn/du-lieu-va-so-lieu-thong-ke/2022/06/thong-cao-bao-chi-ket-qua-khao-sat-muc-song-dan-cu-2021/.
24. The World Bank Group. Official exchange rate (LCU per US$, period average). 2022; https://data.worldbank.org/indicator/PA.NUS.FCRF. Accessed 10 September, 2022.
25. World Bank. GDP per capita (current US$). 2023; https://data.worldbank.org/indicator/NY.GDP.PCAP.CD.

For peer review only

For peer review only - http://bmjopen.bmj.com/site/about/guidelines.xhtml

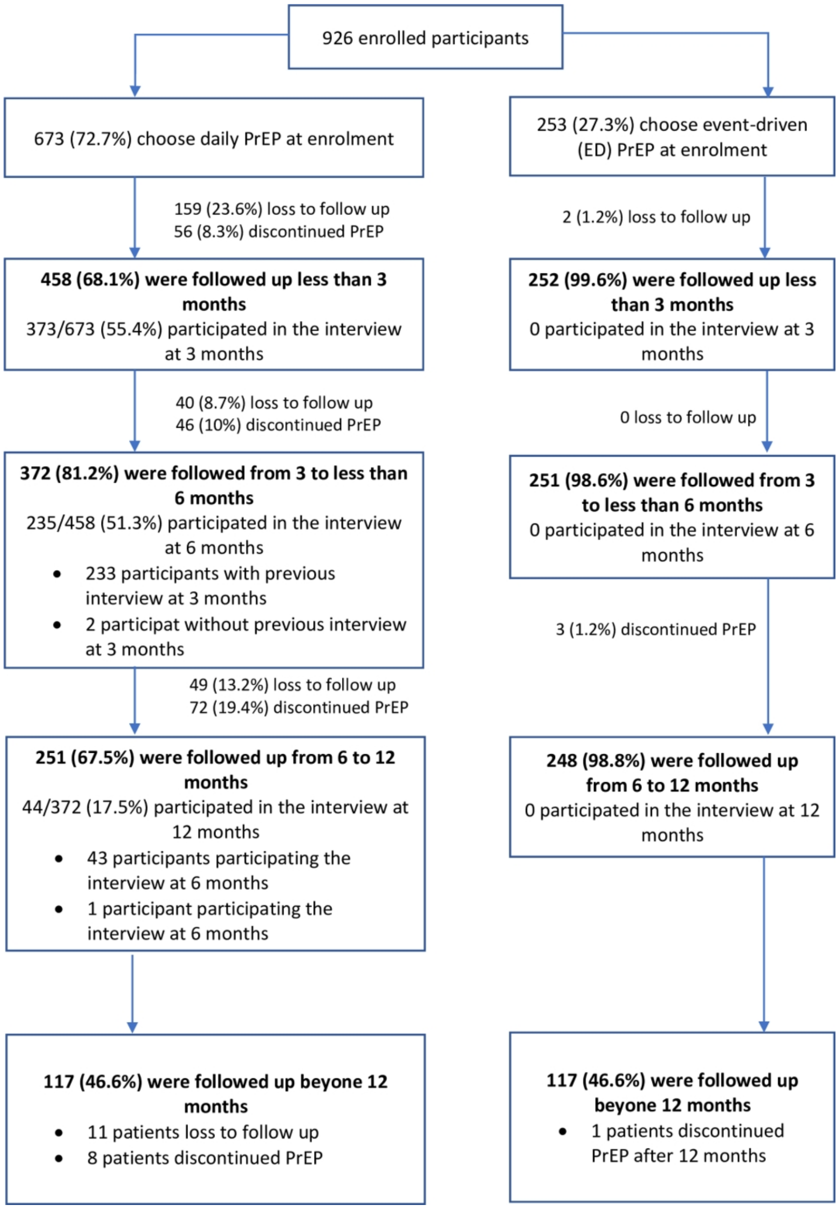

Figure 1. Flowchart of study participants.

154x222mm (300 x 300 DPI)

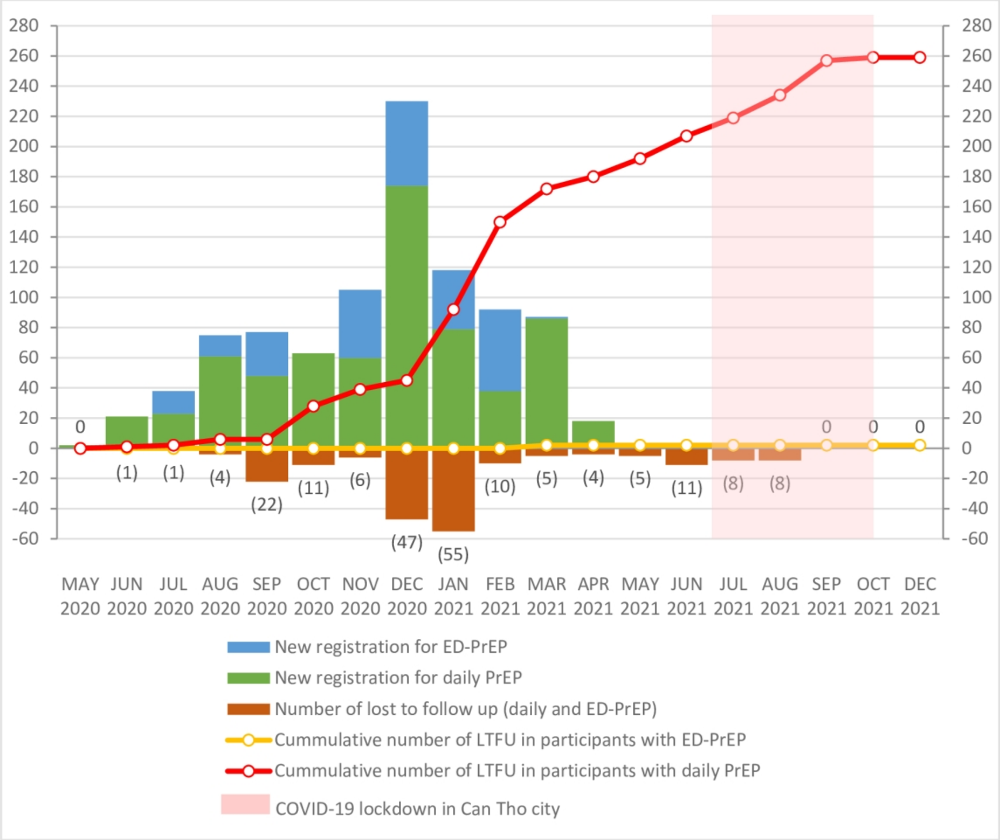

Figure 2. Number of new PrEP initiation and lost to follow up after the study enrolment

159x134mm (300 x 300 DPI)

Supplementary table 1. Baseline characteristic of participants using daily PrEP by the retention (excluding participants who decide to discontinue PrEP due to decreased HIV risk perception)

	All participants (n=491)	Retained in care (n=232)	Lost to follow up (n=259)	P value
**Sex assigned at birth**				
Male	465 (94.7%)	220 (94.8%)	245 (94.6%)	0.908
Female	26 (5.3%)	12 (5.2%)	14 (5.4%)	
**Sexual partners**				
No answer	1 (0.2%)	1 (0.4%)	0 (0.0%)	0.295
Men exclusively	419 (85.3%)	202 (87.1%)	217 (83.8%)	
Men and women	71 (14.5%)	29 (12.5%)	42 (16.2%)	
**HIV exposure within the past 3 days**				
No HIV exposure	488 (99.4%)	231 (99.6%)	257 (99.2%)	0.637
HIV exposure	2 (0.4%)	1 (0.4%)	1 (0.4%)	
No answer	1 (0.2%)	0 (0.0%)	1 (0.4%)	
**Frequency of sexual activity**				
≤ 2 times per week	169 (34.4%)	107 (46.1%)	62 (23.9%)	<0.001
>2 times per week	264 (53.8%)	105 (45.3%)	159 (61.4%)	
No answer	58 (11.8%)	20 (8.6%)	38 (14.7%)	
**Having sex without condom with people who were at risk of HIV within the past 6 months**				
No	130 (26.5%)	53 (22.8%)	77 (29.7%)	0.060
Yes	312 (63.5%)	160 (69.0%)	152 (58.7%)	
No answer	49 (10.0%)	19 (8.2%)	30 (11.6%)	
**Number of sexual partner within the past 6 months**				
1 sexual partner	115 (23.4%)	64 (27.6%)	51 (19.7%)	0.102
At least 2 sexual partners	370 (75.4%)	166 (71.6%)	204 (78.8%)	
No answer	6 (1.2%)	2 (0.9%)	4 (1.5%)	
**Having sex for money or gifts within the past 6 months**				
No	461 (93.9%)	225 (97.0%)	236 (91.1%)	0.017
Yes	21 (4.3%)	6 (2.6%)	15 (5.8%)	
No answer	9 (1.8%)	1 (0.4%)	8 (3.1%)	
**Diagnosis and/or treatment with an STI within the past 6 months**				
No	439 (89.4%)	208 (89.7%)	231 (89.2%)	0.006
Yes	39 (7.9%)	23 (9.9%)	16 (6.2%)	
No answer	13 (2.6%)	1 (0.4%)	12 (4.6%)	
**Sharing needles with other people within the past 6 months**				
No	486 (99.8%)	230 (100.0%)	256 (99.6%)	0.344

For peer review only

Yes	0 (0.0%)	0 (0.0%)	0 (0.0%)	
No answer	1 (0.2%)	0 (0.0%)	1 (0.4%)	
**Used PrEP within the past 6 months**				
No	451 (92.6%)	199 (86.5%)	252 (98.1%)	<0.0001
Yes	33 (6.8%)	31 (13.5%)	2 (0.8%)	
No answer	3 (0.6%)	0 (0.0%)	3 (1.2%)	
**Willingness to pay for PrEP**				
< $4.25/month	100 (20.4%)	44 (19.0%)	56 (21.6%)	0.101
$4.25-$14.89/month	302 (61.5%)	138 (59.5%)	164 (63.3%)	
$14.89-42.55$/month	67 (13.6%)	41 (17.7%)	26 (10.0%)	
>$42.55/month	1 (0.2%)	1 (0.4%)	0 (0.0%)	
Don't want/unable to pay for PrEP	21 (4.3%)	8 (3.4%)	13 (5.0%)	

For peer review only

Supplementary table 3. Concerns about potential barriers to access PrEP at baseline

	All	Daily PrEP	ED-PrEP
Anticipated barrier to PrEP	338 (36.5%)	265 (39.4%)	73 (28.9%)
Concern about side effects	9 (1.0%)	9 (1.3%)	0 (0.0%)
Difficult in managing the time for visiting clinic and drug pick up PrEP	8 (0.9%)	5 (0.7%)	3 (1.2%)
Concerned about stigma/community perception	8 (0.9%)	8 (1.2%)	0 (0.0%)
Concerned about family finding out	6 (0.6%)	6 (0.9%)	0 (0.0%)
Concern about cost	6 (0.6%)	6 (0.9%)	0 (0.0%)
Concerned about stigma/community perception	4 (0.4%)	4 (0.6%)	0 (0.0%)
Difficulty in planning sex in advance	3 (0.3%)	1 (0.1%)	2 (0.8%)
Concerned about carrying the pills	2 (0.2%)	2 (0.3%)	0 (0.0%)
Difficulty in taking the pills 2 hours before sex	1 (0.1%)	1 (0.1%)	0 (0.0%)
Difficulty in remembering to take tablet everyday	1 (0.1%)	0 (0.0%)	1 (0.4%)
Drug storage	1 (0.1%)	1 (0.1%)	0 (0.0%)
Difficulty in managing the time for visiting clinic and drug pick up	1 (0.1%)	0 (0.0%)	1 (0.4%)
Concerned on the comorbidity of asthma	1 (0.1%)	0 (0.0%)	1 (0.4%)
Difficulty in transportation of PrEP	0 (0.0%)	0 (0.0%)	0 (0.0%)
Concerned about partner finding out	0 (0.0%)	0 (0.0%)	0 (0.0%)
Difficulty in remembering to take the following doses	0 (0.0%)	0 (0.0%)	0 (0.0%)
Difficulty in accessing STI, hepatitis testing and/or treatment	0 (0.0%)	0 (0.0%)	0 (0.0%)

For peer review only

Supplementary table 3. Challenges reported by daily PrEP users at 3, 6 and 12 months interviews

	**3 months N= 373**	**6 months N=235**	**12 months N=44**
**Willing to pay for PrEP per month**			

Don't want/unable to pay for PrEP	19 (5.1%)	9 (3.8%)	0 (0.0%)
>$42.55	1 (0.3%)	1 (0.4%)	0 (0.0%)
$14.89-42.55$	45 (12.1%)	36 (15.3%)	21 (47.7%)
$4.25-$14.89	240 (64.3%)	152 (64.7%)	22 (50.0%)
< $4.25	68 (18.2%)	36 (15.3%)	1 (2.3%)
**In the past 3 months, do you use daily PrEP or ED PrEP?**			
Both daily and ED PrEP	17 (4.6%)	3 (1.3%)	0 (0.0%)
Daily PrEP	296 (79.4%)	190 (80.9%)	36 (81.8%)
Any difficulties in taking PrEP medication in the past 3 months	110 (29.5%)	22 (9.4%)	13 (29.5%)
Forgot to take one or more PrEP doses	37 (9.9%)	9 (3.8%)	5 (11.4%)
Fitting PrEP into daily routine (for daily PrEP users)	7 (1.9%)	1 (0.4%)	1 (2.3%)
Unsure when to take PrEP medications	1 (0.3%)	1 (0.4%)	0 (0.0%)
Didn't know what to do about a missed dose	5 (1.3%)	3 (1.3%)	2 (4.5%)
Concerns about interactions with other medications	11 (2.9%)	5 (2.1%)	7 (15.9%)
Concerns about interactions with alcohol	2 (0.5%)	3 (1.3%)	2 (4.5%)
Concerns about interactions with hormonal therapy	1 (0.3%)	1 (0.4%)	0 (0.0%)
Have you had any difficulties accessing PrEP services?	131 (35.1%)	53 (22.6%)	18 (40.9%)
Difficult in managing the time for visiting clinic and drug pick up	37 (9.9%)	18 (7.7%)	6 (13.6%)
Transportation difficulty	11 (2.9%)	13 (5.5%)	13 (29.5%)
Concerned about partner finding out	5 (1.3%)	5 (2.1%)	1 (2.3%)
Concerned about family finding out	32 (8.6%)	12 (5.1%)	2 (4.5%)
Concerned about stigma/community perception	9 (2.4%)	5 (2.1%)	0 (0.0%)
Difficulty in remembering to take tablet everyday	17 (4.6%)	2 (0.9%)	0 (0.0%)
Carrying the pills with me	13 (3.5%)	7 (3.0%)	5 (11.4%)
Concern about side effects	52 (13.9%)	12 (5.1%)	4 (9.1%)
Concern about cost	9 (2.4%)	1 (0.4%)	0 (0.0%)
Difficulty in accessing STI, Hepatitis testing and/or treatment	5 (1.3%)	3 (1.3%)	1 (2.3%)
Health services closed due to Covid-19	33 (8.8%)	21 (8.9%)	16 (36.4%)

For peer review only

Not able to leave home due to Covid-19 lockdown	34 (9.1%)	27 (11.5%)	15 (34.1%)
**Side effects**			
Sleepy	1 (0.3%)	0 (0.0%)	0 (0.0%)
Nausea	12 (3.2%)	1 (0.4%)	1 (2.3%)
Muscle pain	1 (0.3%)	0 (0.0%)	0 (0.0%)
Loss of appetite	2 (0.5%)	0 (0.0%)	0 (0.0%)
Headache	3 (0.8%)	3 (1.3%)	2 (4.5%)
Dry lips	5 (1.3%)	2 (0.9%)	1 (2.3%)
Burning	5 (1.3%)	2 (0.9%)	4 (9.1%)
Acne	0 (0.0%)	0 (0.0%)	2 (4.5%)
Hyperpigmentation	0 (0.0%)	0 (0.0%)	0 (0.0%)
Fatigue	7 (1.9%)	6 (2.5%)	1 (2.3%)
Dizziness	7 (1.9%)	0 (0.0%)	0 (0.0%)
Dry skin	0 (0.0%)	0 (0.0%)	1 (2.3%)

For peer review only

STROBE Statement—checklist of items that should be included in reports of observational studies

	Item No	Recommendation	Page No
**Title and abstract**	1	(*a*) Indicate the study's design with a commonly used term in the title or the abstract	1
		(*b*) Provide in the abstract an informative and balanced summary of what was done and what was found	2
**Introduction**			
Background/rationale	2	Explain the scientific background and rationale for the investigation being reported	4
Objectives	3	State specific objectives, including any prespecified hypotheses	5
**Methods**			
Study design	4	Present key elements of study design early in the paper	5
Setting	5	Describe the setting, locations, and relevant dates, including periods of recruitment, exposure, follow-up, and data collection	5
Participants	6	(*a*) Give the eligibility criteria, and the sources and methods of selection of participants	5
Variables	7	Clearly define all outcomes, exposures, predictors, potential confounders, and effect modifiers. Give diagnostic criteria, if applicable	5
Data sources/ measurement	8*	For each variable of interest, give sources of data and details of methods of assessment (measurement). Describe comparability of assessment methods if there is more than one group	*6*
Bias	9	Describe any efforts to address potential sources of bias	9
Study size	10	Explain how the study size was arrived at	5
Quantitative variables	11	Explain how quantitative variables were handled in the analyses. If applicable, describe which groupings were chosen and why	NA
Statistical methods	12	(*a*) Describe all statistical methods, including those used to control for confounding	6
		(*b*) Describe any methods used to examine subgroups and interactions	6
		(*c*) Explain how missing data were addressed	Fig 11
		(*d*) If applicable, describe analytical methods taking account of sampling strategy	NA
		(*e*) Describe any sensitivity analyses	NA
**Results**			
Participants	13*	(a) Report numbers of individuals at each stage of study—eg numbers potentially eligible, examined for eligibility, confirmed eligible, included in the study, completing follow-up, and analysed	6
		(b) Give reasons for non-participation at each stage	6
		(c) Consider use of a flow diagram	
Descriptive data	14*	(a) Give characteristics of study participants (eg demographic, clinical, social) and information on exposures and potential confounders	6
		(b) Indicate number of participants with missing data for each variable of interest	6
Outcome data	15*	Report numbers of outcome events or summary measures	6

For peer review only

For peer review only - http://bmjopen.bmj.com/site/about/guidelines.xhtml

Main results	16	(*a*) Give unadjusted estimates and, if applicable, confounder-adjusted estimates and their precision (eg, 95% confidence interval). Make clear which confounders were adjusted for and why they were included	7
		(*b*) Report category boundaries when continuous variables were categorized	NA
		(*c*) If relevant, consider translating estimates of relative risk into absolute risk for a meaningful time period	NA
Other analyses	17	Report other analyses done—eg analyses of subgroups and interactions, and sensitivity analyses	7
**Discussion**			
Key results	18	Summarise key results with reference to study objectives	8
Limitations	19	Discuss limitations of the study, taking into account sources of potential bias or imprecision. Discuss both direction and magnitude of any potential bias	9
Interpretation	20	Give a cautious overall interpretation of results considering objectives, limitations, multiplicity of analyses, results from similar studies, and other relevant evidence	9
Generalisability	21	Discuss the generalisability (external validity) of the study results	9
**Other information**			
Funding	22	Give the source of funding and the role of the funders for the present study and, if applicable, for the original study on which the present article is based	8

*Give information separately for exposed and unexposed groups.

Note: An Explanation and Elaboration article discusses each checklist item and gives methodological background and published examples of transparent reporting. The STROBE checklist is best used in conjunction with this article (freely available on the Web sites of PLoS Medicine at http://www.plosmedicine.org/, Annals of Internal Medicine at http://www.annals.org/, and Epidemiology at http://www.epidem.com/). Information on the STROBE Initiative is available at www.strobe-statement.org.

For peer review only

For peer review only - http://bmjopen.bmj.com/site/about/guidelines.xhtml

BMJ Open

Preference and retention of daily and event-driven pre-exposure prophylaxis for HIV prevention: a prospective cohort in Can Tho city, Viet Nam

Journal:	*BMJ Open*
Manuscript ID	bmjopen-2023-075976.R1
Article Type:	Original research
Date Submitted by the Author:	05-Dec-2023
Complete List of Authors:	Nguyen, Van Thi Thuy; World Health Organization, WHO Viet Nam<br>Dat, Vu Quoc; Hanoi Medical University, Department of Infectious Diseases; Hanoi Medical University, Hanoi Medical University Hospital<br>Truc, Huynh Minh; Can Tho city Center for Disease Control<br>Nguyen Anh Thu, Pham; Can Tho city Center for Disease Control<br>Linh, Doan Thi Thuy; Vietnam Administration for HIV/AIDS Control<br>Johnson, Cheryl; World Health Organization, Department of HIV/AIDS<br>Baggaley, R; World Health Organization<br>Phan, Huong; Vietnam Administration of HIV/AIDS control
**Primary Subject Heading**:	HIV/AIDS
Secondary Subject Heading:	Public health
Keywords:	HIV & AIDS < INFECTIOUS DISEASES, Health Services Accessibility, PREVENTIVE MEDICINE

SCHOLARONE™
Manuscripts

I, the Submitting Author has the right to grant and does grant on behalf of all authors of the Work (as defined in the below author licence), an exclusive licence and/or a non-exclusive licence for contributions from authors who are: i) UK Crown employees; ii) where BMJ has agreed a CC-BY licence shall apply, and/or iii) in accordance with the terms applicable for US Federal Government officers or employees acting as part of their official duties; on a worldwide, perpetual, irrevocable, royalty-free basis to BMJ Publishing Group Ltd ("BMJ") its licensees and where the relevant Journal is co-owned by BMJ to the co-owners of the Journal, to publish the Work in this journal and any other BMJ products and to exploit all rights, as set out in our licence.

The Submitting Author accepts and understands that any supply made under these terms is made by BMJ to the Submitting Author unless you are acting as an employee on behalf of your employer or a postgraduate student of an affiliated institution which is paying any applicable article publishing charge ("APC") for Open Access articles. Where the Submitting Author wishes to make the Work available on an Open Access basis (and intends to pay the relevant APC), the terms of reuse of such Open Access shall be governed by a Creative Commons licence – details of these licences and which Creative Commons licence will apply to this Work are set out in our licence referred to above.

Other than as permitted in any relevant BMJ Author's Self Archiving Policies, I confirm this Work has not been accepted for publication elsewhere, is not being considered for publication elsewhere and does not duplicate material already published. I confirm all authors consent to publication of this Work and authorise the granting of this licence.

For peer review only

Preference and retention of daily and event-driven pre-exposure prophylaxis for HIV prevention: a prospective cohort in Can Tho city, Viet Nam

Running Title: Pre-exposure prophylaxis for HIV in Viet Nam

Van Thi Thuy Nguyen[1¥], Vu Quoc Dat[2,3¥*], Huynh Minh Truc[4], Pham Nguyen Anh Thu[4], Doan Thi Thuy Linh[5], Cheryl Johnson[6], Rachel Baggaley[6], Phan Thi Thu Huong[5]

[1] World Health Organization - Viet Nam Country Office, Hanoi, Viet Nam

[2] Department of Infectious Diseases, Hanoi Medical University, Hanoi, Viet Nam

[3] Hanoi Medical University Hospital, Hanoi Medical University, Hanoi, Viet Nam

[4] City Center for Disease Control and Prevention, Can Tho city, Viet Nam

[5] Viet Nam Administration for HIV/AIDS Control, Viet Nam

[6] World Health Organization, Global HIV, Viral hepatitis and STIs programmes, Geneva, Switzerland

[¥] Co-first author

*Corresponding author: Vu Quoc Dat, MD, PhD. Email: datvq@hmu.edu.vn. Postal address: No 1 Ton That Tung Street, Hanoi, Viet Nam.

For peer review only - http://bmjopen.bmj.com/site/about/guidelines.xhtml

Abstract

Objective

Pre-exposure prophylaxis (PrEP) was introduced in Viet Nam in 2017, but data on oral PrEP preference and effective use beyond 3 months are limited. We aimed to evaluate PrEP preferences for PrEP, factors influencing uptake, choice and effective use, as well as barriers to PrEP.

Methods

This is a prospective cohort study in Can Tho, Viet Nam. Participants who were eligible for PrEP and provided informed consent were interviewed at baseline on demographic information, willingness to pay, reasons for choosing their PrEP regimen and the anticipated difficulties in taking PrEP and followed-up at 3 months, 6 months and 12 months after PrEP initiation.

Findings

Between May 2020 and April 2021, 926 individuals at substantial risk for HIV initiated PrEP. Of whom 673 (72.7%) choose daily PrEP and 253 (27.3%) choose event-driven (ED)-PrEP. The majority of participants were men (92.7%) and only 6.8% were women and 0.5% were transgender women. Median participant age was 24 years (IQR 20-28) and 84.7% reported as exclusively same sex-relationship. The three most common reasons for choosing daily PrEP were effectiveness (24.3%) and unplanning for sex (22.9%). Those opting for ED-PrEP also cited effectiveness (22.7%), as well as convenience (18.0%) and easier effective-use 12.0%). Only 7.8% of PrEP users indicated they were unwilling to pay for PrEP and 76.4% would be willing to pay if PrEP were less than US $15 per month. The proportion of user effectively using PrEP at 12 months was 43.1% and 99.2% in daily PrEP and ED-PrEP users, respectively.

Conclusions

Event-driven PrEP was preferred by more than a quarter of 23.5% of the participants and there was little concern about potential adverse events. High rates of effective use were reported by ED-PrEP users. Future research to inform implementation of PrEP in Viet Nam is needed to develop ways of measuring adherence to ED-PrEP more accurately and to understand and address difficulties in taking daily PrEP use.

Keywords: PrEP, pre-exposure prophylaxis, daily PrEP, event-driven PrEP, on-demand PrEP, MSM, retention, Viet Nam

For peer review only - http://bmjopen.bmj.com/site/about/guidelines.xhtml

Strengths and limitations of this study

- We conducted the first study on preference, retention and factors associated with these in PrEP use in Viet Nam.
- The major limitations related to the study design of single center
- The self-statement of adherence among ED-PrEP users in this study could contribute to overestimate of the retention.

For peer review only

For peer review only - http://bmjopen.bmj.com/site/about/guidelines.xhtml

Introduction

As of December 2020, Viet Nam reported that there were 215,220 people with HIV, in which there were 12,200 new HIV infections and 1,681 AIDS-related deaths in 2020 (1). The HIV epidemic in Viet Nam is concentrated in key populations including people who inject drugs (PWID), men who have sex with men (MSM) and female sex workers (FSWs). It is estimated that there are approximately 200,000 MSM in Viet Nam(2). In recent years, HIV prevalence has increased in the MSM population, from 5.1% in 2015 to 13.3% in 2020, while prevalence was stable in PWID populations (12.7% in 2019) and FSWs (3.1% in 2020) (1).

Since 2016, the World Health Organization (WHO) has recommended oral pre-exposure prophylaxis (PrEP) to further reduce new infections among populations where HIV incidence and risk is high. Following this guidance, between June and December 2017, Viet Nam updated their national guidelines and started initial PrEP implementation in Hanoi and Ho Chi Minh city. Since then, PrEP implementation in Viet Nam has continued to expand and as of August of 2021, there were nearly 32,000 persons using PrEP across 200 clinics in nearly half of all provinces in the country(3). Current national guidelines recommend daily-PrEP (tenofovir disoproxil fumarate (TDF) co-formulated with emtricitabine (FTC) or lamivudine (3TC)) for populations at substantial risk and event-driven PrEP (ED-PrEP) (TDF/XTC) for MSM who have less frequent sex (<2 times per week) (4).

While oral PrEP continues to expand and be an effective option for many, recent evidence has highlighted that more differentiated service delivery options are needed. In particular, ED-PrEP provides an effective option which removes the need for daily doses and for use before and after high risk sex. Among MSM, ED-PrEP has been shown to reduce HIV transmission by up to 86% (5). Studies have also shown that MSM may often prefer ED-PrEP over daily oral PrEP because of its convenience. In a US-survey, 74.3% of MSM who were hesitant to start oral daily PrEP indicated that they would be more willing to try oral ED-PrEP (6). In Thailand some PrEP users considered daily regimens the easiest to use, as it could be incorporated into daily routines and did not require planning for sex. These men expressed concerns, however, about the long-term safety and affordability of daily oral dosing (7). Study participants appreciated oral ED-PrEP for minimizing drug exposure and potential adverse events. They considered ED-PrEP an attractive choice for MSM who had infrequent sex, were able to plan for sex, and had the ability to take the post-sex dose (7).

Despite the potential benefits of ED-PrEP, it is little known about preference and uptake of ED-PrEP among MSM in Viet Nam. Thus, this study aims to assess both preferences as well as actual uptake and continuation of oral daily and ED-PrEP among MSM in Viet Nam to inform future

For peer review only - http://bmjopen.bmj.com/site/about/guidelines.xhtml

programming. In addition, difficulties related to PrEP uptake and continuation including COVID-19-related issues were explored to inform future differentiated PrEP service delivery models.

Methods

Study design and participants

We conducted a prospective study in all 11 PrEP clinics in Can Tho which has the highest HIV prevalence among MSM (22.7%)(2). MSM were referred to the PrEP clinics from community-based HIV testing led by MSM groups or via self-referral. All clinics were integrated with HIV testing and/or ART services. PrEP eligibility was evaluated following the national guideline: (1) confirmed HIV-negative status, (2) no signs and symptoms of acute HIV infection and (3) at substantial risk for HIV infection within past 6 months. We defined substantial risk as any of the following: individual engaged in condomless anal or vaginal sex, having at least 2 sexual partners, reported sexual partner with substantial risk for HIV infection, or having a sexual partner with HIV but not currently on ART or with unknown/detectable viral load (>200 copies/ml), who had been previously diagnosed with a sexually transmitted infection (STI), and who reported having multiple courses of PEP and continued sexual risk behaviour. Only eligible participants aged 16 years and over who agreed to participate and provide written informed consent were recruited for the study.

Study procedure and data collection

In the community-based setting, PrEP screening and offering different PrEP regimen is part of HIV post-test counselling (HTC) (8). Clients who were interested in PrEP will be referred to a PrEP clinic. At the PrEP clinics, clients were evaluated based on their behavioural risk to assess PrEP eligibility. ED-PrEP were offered for MSM who have infrequent sex (≤ 2 times per week on average) and are usually able to plan for sex at least two hours in advance, or who can delay sex for at least two hours or their own preference of ED-PrEP. During screening, the clinic staff explained what PrEP is, the benefits and differences between daily PrEP and ED-PrEP and let the client decide. After the clients chose their preferred PrEP regimen, they were invited to provide informed consent and participate in the study and provide written informed consents. Daily PrEP regimens were offered based on the availability of the antiretroviral including tenofovir disoproxil fumarate/emtricitabine (TDF/FTC) or tenofovir disoproxil fumarate/lamivudine (TDF/3TC) or TDF. ED- PrEP regimens were offered as TDF/FTC or TDF/3TC

National guidelines recommend follow-up with all PrEP clients at health facilities starting with 1-2 months after PrEP initiation and then quarterly thereafter. We used a questionnaire consisting of six questions on willingness to pay (closed-end questions. Willingness to pay estimates were reported in 2,000 Vietnam Dong (VND) during the interview and converted to US dollar (2021) for this analysis

For peer review only - http://bmjopen.bmj.com/site/about/guidelines.xhtml

(1 US$ = 23,529 Viet Nam Dong). Semi-structured interviews were used to understand potential barriers to PrEP use. PrEP users were monitored following Viet Nam Ministry of Health's guidelines including HIV testing and continuation. Continuation of PrEP was defined if PrEP users who come back to pick up drugs (for daily PrEP) or self-reported to adherence (for ED-PrEP) at the corresponding following up visits after initiation at 3-, 6- and 12-month visits.

Data Analysis

Participants' responses to the open-ended questions were coded by an independent investigator (VQD). Statistical analysis was performed using the IBM SPSS Statistics for Windows, Version 27.0 (Armonk, NY: IBM Corp). We used conventional descriptive statistics to summarise the characteristics of the study's participants and their views on PrEP. Effective use was calculated by dividing the number of PrEP users retained at 3, 6 and 12 months of PrEP by the total number of clients enrolled in PrEP study and multiplying by 100. Multivariable logistic regression was used to estimate adjusted odd ratio (aOR) and 95% CIs for PrEP retention by selected baseline characteristics. P-value < 0.05 were considered statistically significant.

Ethics

The study was approved by Hanoi Medical University Institutional Ethical Review Board (IRB VN01.001/IRB 0000312/FWA 00004148), and WHO Wester Pacific Regional Office Ethical Review Committee (2020.4.VTN.1.HSI).

Patient and public involvement

No patient or public involvement.

Results

Between May 2020 and Apr 2021, we enrolled 926 clients of whom 253 (27.3%) choose ED-PrEP and 673 (72.7%) choose daily PrEP at enrolment. Participants were follow-up until 31 December 2022. Table 1 and Supplementary table 1 show the characteristics of enrolled participants. The median age was 24 years (IQR 20-28) and ranging from 16 to 51 years. Twelve participants were under 18 years old (1.3%). The majority of men participants reported their exclusive sex with men (784/926 or 84.7%) and there was no significant difference in age and gender identity between groups of daily PrEP and ED-PrEP.

At baseline, 94.1% (871/926) participants responded to an open-ended question on the reasons that determined their PrEP preference after the consultation with the clinic staff. The response rate for reporting the reasons PrEP preference was not significant between the groups of daily PrEP (94.8%; 638/673) and ED-PrEP (233/253 or 91.1%). The most common reasons for choosing daily-PrEP were

For peer review only - http://bmjopen.bmj.com/site/about/guidelines.xhtml

For peer review only

the effectiveness of PrEP in preventing HIV infection (155/638 or 24.3%), no need to plan for sex in advance (146/638 or 22.9%) and easy for for longer-term use (121/638 or 19.0%). Among participants who chose ED-PrEP, the most common reasons were effectiveness (53/233 or 22.7%), convenience (42/233 or 18%) and easy for adherence (28/233 or 12.0%). The detailed reasons for PrEP preference at baseline was showed in the Table 2. Among 338/926 (36.5%) participants who anticipated PrEP barriers, only 22/338 (6.5%) (17/265 preferred daily PrEP and 5/73 preferred ED-PrEP) expressed the specific concerns on PrEP (Supplementary table 2).

Regarding the willingness to pay for PrEP, most (76.4%) were willing to pay for PrEP if less than US $15 per month, while some (7.8%) said they would not pay or felt they were unable to pay for PrEP at any cost. The proportions of clients willing to pay at different prices were statistically different between daily and ED-PrEP groups (Table 1).

The median follow-up time was 284 days (IQR 102–367) among 926 participants who initiated PrEP, 214 days (IQR 60-323) in participants choosing daily PrEP and 363 days (IQR 319-389) in participants choosing ED-PrEP. By the end of the study, 261/926 (28.2%) patients were lost to follow- up, 186/926 (20.1%) discontinued and 479/926 (51.7%) were on PrEP (Figure 1). Much of the loss to follow-up occurred within the first 3 months of enrolment (159/261 or 60.9%) and among those taking daily oral PrEP (259/261 or 99.2%). The overall retention rates at 3, 6 and 12 months in the daily PrEP group were 72.6% (439/605), 64.5% (363/563) and 43.1% (150/198), respectively with the median time of lost to follow-up of 60 days. The retention rates in the ED-PrEP group were 99.2% (251/253) at 3 and 6 months and 99.4% (158/159) at 12 months. Of 186 participants who discontinued PrEP, reasons reported for discontinuation were that they were no longer sexually active (87/186 or 46.8%), moving to a new place (85/186 or 45.7%), were diagnosed with HIV (seroconversion) (7/186 or 3.8%), had different user preferences, had concerns about medication related toxicities, were diagnosed with HBV (each of 2/185 or 1.1%) or were affected due to COVID-19-related restrictions (1/186 or 0.5%).

Of participants who started daily PrEP and completed the interview, the proportions of participants reporting any PrEP side effects were 32/341 (8.6%) at 3 months, 12/235 (5.1%) at 6 months and 5/44 (11.4%) at 12 months. The detail of reported side effects was listed in the Supplementary table 3.

During COVID-19 pandemic, Can Tho city was locked down due to COVID-19 outbreak (e.g from July – October 2021). However, as shown in the Figure 2, a larger percentage of participants discontinued PrEP before the lockdown period. There were only 40/261 (15.3%) participants who lost to follow up within 4 months of lockdown and before of the study completion.

For peer review only - http://bmjopen.bmj.com/site/about/guidelines.xhtml

We also carried out multivariable logistic regression to determine factors associated with PrEP retention among daily PrEP participants (continued daily PrEP group vs lost-to-follow-up group). The results indicate that factors associated with daily PrEP continuation were less frequency of sex (less than twice per week) (aOR 2.199, 95%CI 1.306-3.702,), having sex without condom with people who were at risk of HIV within the past 6 months (aOR 1.991, 95%CI 1.180-3.362), PrEP use within the past 6 mnths (aOR 13.568, 95%CI 3.015-61.071) and no anticipation of barriers to PrEP use (aOR 1.721, 95%CI 1.042-2.842) (Table 3). Due to small number of lost to follow-up among ED-PrEP users, we were unable to execute multivariable Cox regression for this group.

Discussion

This study is the first prospective study in Viet Nam to explore both PrEP preferences and use, as well as effective use and factors associated with PrEP continuation. We found that among individuals who were eligible for PrEP, more than a quarter preferred ED-PrEP over daily PrEP. The proportion of the MSM who preferred ED-PrEP in our study was similar to the studies in high income countries, including Belgium (23.4%-23.5%) (9, 10), the Netherland (26.7%-27.3%)(10, 11) and Australia (~20%)(12, 13) but was lower than countries such as France (49.5%)(14), Taiwan (56%)(15), China (57.1%)(16) and others in West Africa (72.1%-74%)(17, 18). We reported a great variety of individual factors determine the choices for their PrEP regimens, mostly related to the participants' perceptions of PrEP efficacy in prevention of HIV transmission, safety, perceived adherence and convenience. In a qualitative study in 857 MSM on daily PrEP and 301 MSM on ED-PrEP in Netherlands, preference of oral PrEP was reported to include frequency of sex, expected adherence, perceived safety, efficacy and burden of the pills and anticipated side effects(19). In a prospective study in 1000 MSM who use oral PrEP in China, the multivariable marginal effect analysis show that factors associated with an increased preference for daily versus ED-PrEP were currently being married to or living with a female (adjusted marginal effect = −0.146 [95% CI: −0.230, −0.062], *p* = 0.001), number of male sexual partners in the previous six months (adjusted marginal effect = 0.003, 95% CI: 0.000- 0.005], *p* = 0.034) and a subjective assessment of being very high risk of HIV infection (adjusted marginal effect size = 0.105 [95% CI: 0.012, 0.198], *p* = 0.027)(16).

For PrEP effective use, we found that those on ED-PrEP had greater continuation rate compared to those opting for daily oral PrEP at 12 months (99.2% and 43.1%, p<0.001 respectively). Daily PrEP retention has been reported in different studies with large variations by study design, PrEP delivery approaches and countries. The proportion of retention at 12 months ranged from 43% in a study of 5,583 MSM from 2012 to 2017 in 6 clinical sites in United State (20), 72.3% in a cohort of 1347 PrEP users in Belgium between 2017 and 2020(21), 83% in 450 MSM in Brazil between 2014 and

For peer review only - http://bmjopen.bmj.com/site/about/guidelines.xhtml

2016(22) to 91.8% in a study of 400 MSM in 12 urban US cities in 2013 (23). In a randomized control trial with 119 MSM in Hong Kong, the oral daily and ED PrEP retention at 32 week was 86% and 87% respectively(24). Proportion of retention to ED-PrEP among Thai MSM aged 15-19 years was 88.9%, 95% CI: 73.9-96.9%) at 6 months(25). The definition of retention for PrEP was varied and made it difficult to compare the proportion of retention across the studies. It was conventionally defined as the return for follow-up every 3 months(26) or attendance at a specific timepoint (eg. 3, 6 and 12 months) with a time window (±30 days) while clients may not attend a follow up visit with a precise intervals(27), especially in case of ED-PrEP. In addition, concepts of retention are changing and that people come during periods of risk and have different needs. Thus, effective use of PrEP is increasingly being used while adherence and retention are not. Our findings suggest more diverse and flexible PrEP models might lead to better use and engagement from clients. These results can also be leveraged to improve oral daily PrEP by making services and follow-up more differentiated including use of HIVST use for PrEP continuation. We found that the majority of MSM in Can Tho were willing to pay for PrEP (92.2%). However, 82.8% (707/854) participants in our study indicated that their willingness to pay was low (<15 US$/month) (the average income per person in Mekong delta was 3,713,000 VND(28) or approximately US160.3, at the exchange rate US$1=23,159.8 VND in 2021(29)) while 65% of respondents in Thailand willing to pay US$25 (monthly average income per person was US$478.1 in 2012(30)) and 88.9% of respondents in China would like to pay > US$14 per month for PrEP (monthly average income per person was US$ 867.3 in 2020(30)).

We noticed that the number of new registration for PrEP and the number of lost-to-follow up were highest between December 2021 and January 2022. In Viet Nam, December was designated for the HIV action month with many events promoted for HIV interventions including PrEP, which may have been related to the increase in the new registration and also high rate of lost-to-follow up thereafter.

Limitation

Our limitation in this study was the patients were recruited from a single province; thus, the study site is purposely selected and may not represent all PrEP users in Viet Nam. The self-report on challenges during PrEP taking could be recall bias. In addition, we didn't included information on re-starting PrEP or switching from daily PrEP to ED-PrEP in this analysis which may cause a potential bias of underestimate the retention rate. Also, self-statement of adherence among ED-PrEP users could contribute to overestimate of the retention in this group.

For peer review only - http://bmjopen.bmj.com/site/about/guidelines.xhtml

Conclusion

Individuals at substantial risk for HIV especially MSM in Can Tho, Viet Nam were motivated to choose PrEP by their beliefs about the safety, efficacy and, frequency of sex and expected adherence with little concerns about side effects and specific barriers to use PrEP. ED-PrEP was desirable and achieved high levels of effective use in this cohort study, but with low willingness to pay. ED-PrEP is desirable and should be offered as an option to expand access and prevent new infections in Viet Nam. Further research is needed to provide more insights, particularly on loss to follow up and implementation of more flexible PrEP delivery models.

Data availability statement

Data are available upon reasonable request.

Funding

The study was funded by Viet Nam - WHO Country Office. Grant number: NA,

Contributors

VNTT, PTTH conceived and designed the study. VQD analysed the data and wrote the first draft of the report. VTTN, PTTH, HMT, PNAT, and DTTL involved to the acquisition and interpretation of data. VTTN, CJ, RB, SD and PTTH reviewed the manuscript and provided substantial contribution. All authors contributed to the manuscript finalization and approved the final report.

Competing interests

We declare no competing interest.

Acknowledgments

We are very grateful to the health staff at Can Tho CDC and the participating PrEP clinics in Can Tho who contributed to the implementation of the study.

For peer review only - http://bmjopen.bmj.com/site/about/guidelines.xhtml

Table 1. Characteristic of participants by the initial PrEP preference

Characteristics	All participants (n=926)	Participants preferred a daily PrEP regimen (n=673)	Participants preferred ED PrEP regimen (n=253)	p value
**Gender identity**				<0.001
Male	858 (92.7%)	608 (90.3%)	250 (98.8%)	
Female	63 (6.8%)	61 (9.1%)	2 (0.8%)	
Trans female	5 (0.5%)	4 (0.6%)	1 (0.4%)	
**Age (median, IQR) (years)**	24 (20-28)	24 (20-28)	23 (21-27)	
**Sexual partners**				0.568
No answer	3 (0.3%)	3 (0.4%)	0 (0.0%)	
Men exclusively	784 (84.7%)	569 (84.5%)	215 (85.0%)	
Men and women	139 (15%)	101 (15%)	38 (15%)	
**HIV exposure within the past 3 days**				0.224
No HIV exposure	915 (98.8%)	667 (99.1%)	248 (98.0%)	
HIV exposure	10 (1.1%)	5 (0.7%)	5 (2.0%)	
No answer	1 (0.1%)	1 (0.1%)	0 (0.0%)	
**Frequency of sexual activity**				<0.001
≤ 2 times per week	279 (32.1%)	228 (37.0%)	51 (20.2%)	
>2 times per week	561 (64.6%)	366 (59.4%)	195 (77.4%)	
No answer	28 (3.2%)	22 (3.6%)	6 (2.4%)	
**Having sex without condom with people who were at risk of HIV within the past 6 months**				<0.001
No	352 (38.0%)	183 (27.2%)	169 (66.8%)	
Yes	502 (54.2%)	424 (63.0%)	78 (30.8%)	
No answer	72 (7.8%)	66 (9.8%)	6 (2.4%)	
**Number of sexual partners within the past 6 months**				<0.001
1 sexual partner	205 (22.1%)	171 (25.4%)	36 (14.2%)	
At least 2 sexual partners	703 (75.9%)	495 (73.6%)	208 (82.2%)	

For peer review only

For peer review only - http://bmjopen.bmj.com/site/about/guidelines.xhtml

No answer	16 (1.7%)	7 (1.0%)	9 (3.6%)	
**Having sex for money or gifts within the past 6 months**				0.002
No	835 (90.2%)	619 (92.0%)	216 (85.4%)	
Yes	60 (6.5%)	32 (4.8%)	28 (11.1%)	
No answer	31 (3.3%)	22 (3.3%)	9 (3.6%)	
**Diagnosis and/or treatment with an STI within the past 6 months**				<0.001
No	793 (85.6%)	597 (88.7%)	196 (77.5%)	
Yes	103 (11.1%)	51 (7.6%)	52 (20.6%)	
No answer	30 (3.2%)	25 (3.7%)	5 (2.0%)	
**Sharing needles with other people within the past 6 months**				0.387
No	917 (99.5%)	664 (99.3%)	253 (100.0%)	
Yes	2 (0.2%)	2 (0.3%)	0 (0.0%)	
No answer	3 (0.3%)	3 (0.4%)	0 (0.0%)	
**Used PrEP within the past 6 months**				0.001
No	874 (94.8%)	623 (93.1%)	251 (99.2%)	
Yes	44 (4.8%)	42 (6.3%)	2 (0.8%)	
No answer	4 (0.4%)	4 (0.6%)	0 (0.0%)	
**Willingness to pay for PrEP**				<0.001
< US$4.25/month	196 (21.2%)	130 (19.3%)	66 (26.1%)	
US$4.25-US$14.89/month	511 (55.2%)	407 (60.5%)	104 (41.1%)	
US$14.89-US$42.55/month	145 (15.7%)	91 (13.5%)	54 (21.3%)	
>US$42.55/month	2 (0.2%)	2 (0.3%)	0 (0.0%)	
Don't want/unable to pay for PrEP	72 (7.8%)	43 (6.4%)	29 (11.5%)	

For peer review only

For peer review only - http://bmjopen.bmj.com/site/about/guidelines.xhtml

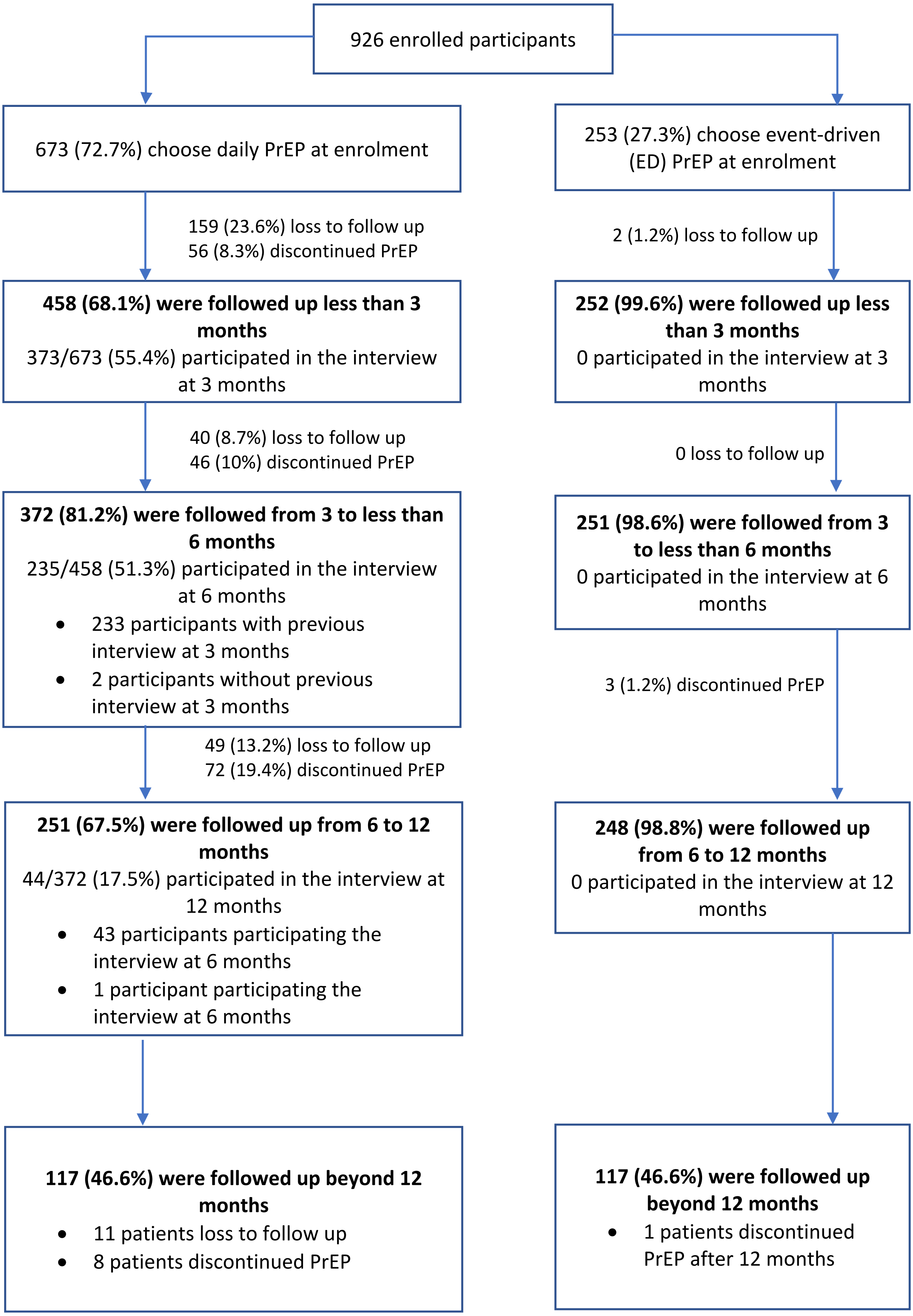
926 enrolled participants
673 (72.7%) choose daily PrEP at enrolment
253 (27.3%) choose event-driven (ED) PrEP at enrolment
159 (23.6%) loss to follow up
56 (8.3%) discontinued PrEP
2 (1.2%) loss to follow up
458 (68.1%) were followed up less than 3 months
373/673 (55.4%) participated in the interview at 3 months
252 (99.6%) were followed up less than 3 months
0 participated in the interview at 3 months
40 (8.7%) loss to follow up
46 (10%) discontinued PrEP
0 loss to follow up
372 (81.2%) were followed from 3 to less than 6 months
235/458 (51.3%) participated in the interview at 6 months
233 participants with previous interview at 3 months
2 participants without previous interview at 3 months
251 (98.6%) were followed from 3 to less than 6 months
0 participated in the interview at 6 months
3 (1.2%) discontinued PrEP
49 (13.2%) loss to follow up
72 (19.4%) discontinued PrEP
251 (67.5%) were followed up from 6 to 12 months
44/372 (17.5%) participated in the interview at 12 months
43 participants participating the interview at 6 months
1 participant participating the interview at 6 months
248 (98.8%) were followed up from 6 to 12 months
0 participated in the interview at 12 months
117 (46.6%) were followed up beyond 12 months
11 patients loss to follow up
8 patients discontinued PrEP
117 (46.6%) were followed up beyond 12 months
1 patients discontinued PrEP after 12 months

Figure 1. Flowchart of study participants.

For peer review only

For peer review only - http://bmjopen.bmj.com/site/about/guidelines.xhtml

Table 2. Reasons for choosing PrEP at baseline

Reasons	All participants	Daily PrEP	ED-PrEP
**Number of sex partners**			
Few sex partners	25 (2.9%)	25 (3.9%)	0 (0.0%)
Multiple sex partners	72 (8.3%)	71 (11.1%)	1 (0.4%)
**Frequency of sex**			
Having less frequent sex	19 (2.2%)	7 (1.1%)	12 (5.2%)
Having more frequent sex	104 (11.9%)	96 (15.0%)	8 (3.4%)
Effectiveness of PrEP	208 (23.9%)	155 (24.3%)	53 (22.7%)
Unplanning for sex	155 (17.8%)	146 (22.9%)	9 (3.9%)
Easy to remember for taking PrEP or adherence	149 (17.1%)	121 (19.0%)	28 (12.0%)
Convenience	79 (9.1%)	37 (5.8%)	42 (18.0%)
Self protection from HIV	60 (6.9%)	38 (6.0%)	22 (9.4%)
No specify	60 (6.9%)	45 (7.1%)	15 (6.4%)
Safe	52 (6.0%)	37 (5.8%)	15 (6.4%)
Match the personal risk	50 (5.7%)	45 (7.1%)	5 (2.1%)
Staying with partner	33 (3.8%)	33 (5.2%)	0 (0.0%)
Having HIV positive sexual partner	29 (3.3%)	28 (4.4%)	1 (0.4%)
Wanting to try	27 (3.1%)	1 (0.2%)	26 (11.2%)
Taking less pills	24 (2.8%)	15 (2.4%)	9 (3.9%)
Self-esteem	20 (2.3%)	20 (3.1%)	0 (0.0%)
Having vaginal sex	18 (2.1%)	18 (2.8%)	0 (0.0%)
Don't want to use condom	17 (2.0%)	8 (1.3%)	9 (3.9%)
Free of charge	16 (1.8%)	4 (0.6%)	12 (5.2%)
Afraid of condom broken	10 (1.1%)	0 (0.0%)	10 (4.3%)
Afraid of HIV infection	7 (0.8%)	2 (0.3%)	5 (2.1%)
Want to have a child	3 (0.3%)	3 (0.5%)	0 (0.0%)
Partner takes daily PrEP	3 (0.3%)	1 (0.2%)	2 (0.9%)
HBV infection	3 (0.3%)	3 (0.5%)	0 (0.0%)
Protect the family	2 (0.2%)	2 (0.3%)	0 (0.0%)
New programme	2 (0.2%)	0 (0.0%)	2 (0.9%)
Doubt about their partners fidelity	2 (0.2%)	1 (0.2%)	1 (0.4%)
Having oral sex	1 (0.1%)	1 (0.2%)	0 (0.0%)
Frequent exposure to blood	1 (0.1%)	1 (0.2%)	0 (0.0%)
Check for the body tolerance	1 (0.1%)	1 (0.2%)	0 (0.0%)

For peer review only

For peer review only - http://bmjopen.bmj.com/site/about/guidelines.xhtml

Figure 2. Number of new PrEP initiation and lost to follow up after the study enrolment

For peer review only

For peer review only - http://bmjopen.bmj.com/site/about/guidelines.xhtml

Table 3. Multivariate logistic regression predicting the retention in daily PrEP in Can Tho (*n* = 414)

Variables	Adjusted odd ratio (95%CI)	P value
**Age (1-yr. increment)**	0.967 (0.934-1.002)	0.065
**Gender identity**		
Male	1	
Female/trans female	1.932 (0.896-4.170)	0.093
**Sexual partners**		
Men and women	1	
Men exclusively	1.720 (0.919-3.218)	0.090
**Frequency of sexual activity**		
>2 times per week	1	
≤ 2 times per week	2.199 (1.306-3.702)	**0.003**
No answer	2.130 (0.927-4.897)	0.075
**Having sex without condom with people who were at risk of HIV within the past 6 months**		
No	1	
Yes	1.991 (1.180-3.362)	**0.010**
**Number of sexual partners within the past 6 months**		
≥2 partners	1	
<2 partners	1.225 (0.722-2.077)	0.452
**Having sex for money or gifts within the past 6 months**		
Yes	1	
No	3.259 (0.742-14.318)	0.118
**Diagnosis and/or treatment with an STI within the past 6 months**		
No	1	
Yes	0.795 (0.325-1.944)	0.615
**Used PrEP within the past 6 months**		
No	1	
Yes	13.568 (3.015-61.071)	**0.001**
**Willingness to pay for PrEP**		
Don't want/unable to pay for PrEP	1	
Willing to pay	0.832 (0.275-2.517)	0.744
**Anticipated barrier to PrEP (1)**		
No	1	
Yes	1.721 (1.042-2.842)	**0.034**

For peer review only - http://bmjopen.bmj.com/site/about/guidelines.xhtml

REFERENCES

1. Ministry of Health. Report on HIV/AIDS prevention in 2020. Hanoi; 2021 4 Feb. Report No.: 124/BC-BYT.
2. The Joint United Nations Programme on HIV and AIDS (UNAIDS), Vietnam Administration of AIDS Control (VAAC). A review of HIV prevention in Vietnam 2019. 2019.
3. Vietnam Administration of AIDS Control (VAAC). Interim guidance on implementation of PrEP in students. 2022.
4. Ministry of Health. Guidelines for the care and treatment of people living with HIV/AIDS 2021.
5. Molina JM, Charreau I, Spire B, Cotte L, Chas J, Capitant C, et al. Efficacy, safety, and effect on sexual behaviour of on-demand pre-exposure prophylaxis for HIV in men who have sex with men: an observational cohort study. Lancet HIV. 2017;4(9):e402-e10.
6. Beymer MR, Gildner JL, Holloway IW, Landovitz RJ. Acceptability of Injectable and On-Demand Pre-Exposure Prophylaxis Among an Online Sample of Young Men Who Have Sex with Men in California. LGBT Health. 2018;5(6):341-9.
7. Chemnasiri T, Varangrat A, Amico KR, Chitwarakorn A, Dye BJ, Grant RM, et al. Facilitators and barriers affecting PrEP adherence among Thai men who have sex with men (MSM) in the HPTN 067/ADAPT Study. AIDS Care. 2020;32(2):249-54.
8. Viet Nam Authority for HIV/AIDS Control. Guidance for implemenmentation of pre-exposure prophylaxis (PrEP). Hanoi: Viet Nam Authority for HIV/AIDS Control,; 2020.
9. Reyniers T, Nostlinger C, Laga M, De Baetselier I, Crucitti T, Wouters K, et al. Choosing Between Daily and Event-Driven Pre-exposure Prophylaxis: Results of a Belgian PrEP Demonstration Project. J Acquir Immune Defic Syndr. 2018;79(2):186-94.
10. Jongen VW, Reyniers T, Ypma ZM, Schim van der Loeff MF, Davidovich U, Zimmermann HM, et al. Choosing event-driven and daily HIV pre-exposure prophylaxis - data from two European PrEP demonstration projects among men who have sex with men. Journal of the International AIDS Society. 2021;24(8):e25768.
11. Coyer L, van den Elshout MAM, Achterbergh RCA, Matser A, Schim van der Loeff MF, Davidovich U, et al. Understanding pre-exposure prophylaxis (PrEP) regimen use: Switching and discontinuing daily and event-driven PrEP among men who have sex with men. EClinicalMedicine. 2020;29-30:100650.
12. Vaccher SJ, Gianacas C, Templeton DJ, Poynten IM, Haire BG, Ooi C, et al. Baseline Preferences for Daily, Event-Driven, or Periodic HIV Pre-Exposure Prophylaxis among Gay and Bisexual Men in the PRELUDE Demonstration Project. Front Public Health. 2017;5:341.
13. C. Chan MH, T.R. Broady, J. MacGibbon, L. Mao,, G. Prestage BW, B. Clifton, B.R. Bavinton. Increased use of event-driven PrEP during COVID-19 in Australia: results from behavioural surveillance 2019-2021. The 12th IAS Conference on HIV Science (IAS 2023); Munich, Germany2023.
14. Molina JM, Ghosn J, Assoumou L, Delaugerre C, Algarte-Genin M, Pialoux G, et al. Daily and on-demand HIV pre-exposure prophylaxis with emtricitabine and tenofovir disoproxil (ANRS PREVENIR): a prospective observational cohort study. Lancet HIV. 2022;9(8):e554-e62.
15. Wu HJ, Wen-Wei Ku S, Chang HH, Li CW, Ko NY, Strong C. Imperfect adherence in real life: a prevention-effective perspective on adherence to daily and event-driven HIV pre-exposure prophylaxis among men who have sex with men - a prospective cohort study in Taiwan. Journal of the International AIDS Society. 2021;24(5):e25733.
16. Zhang J, Xu JJ, Wang HY, Huang XJ, Chen YK, Wang H, et al. Preference for daily versus on-demand pre-exposure prophylaxis for HIV and correlates among men who have sex with men: the China Real-world Oral PrEP Demonstration study. Journal of the International AIDS Society. 2021;24(2):e25667.

For peer review only - http://bmjopen.bmj.com/site/about/guidelines.xhtml

17. Laurent C, Dembele Keita B, Yaya I, Le Guicher G, Sagaon-Teyssier L, Agboyibor MK, et al. HIV pre-exposure prophylaxis for men who have sex with men in west Africa: a multicountry demonstration study. Lancet HIV. 2021;8(7):e420-e8.
18. Laurent C, Yaya I, Cuer B, Sagaon-Teyssier L, Mensah E, Dah TTE, et al. Human Immunodeficiency Virus Seroconversion Among Men Who Have Sex With Men Who Use Event-Driven or Daily Oral Pre-Exposure Prophylaxis (CohMSM-PrEP): A Multi-Country Demonstration Study From West Africa. Clin Infect Dis. 2023;77(4):606-14.
19. Zimmermann HM, Eekman SW, Achterbergh RC, Schim van der Loeff MF, Prins M, de Vries HJ, et al. Motives for choosing, switching and stopping daily or event-driven pre-exposure prophylaxis - a qualitative analysis. Journal of the International AIDS Society. 2019;22(10):e25389.
20. Rusie LK, Orengo C, Burrell D, Ramachandran A, Houlberg M, Keglovitz K, et al. Preexposure Prophylaxis Initiation and Retention in Care Over 5 Years, 2012-2017: Are Quarterly Visits Too Much? Clin Infect Dis. 2018;67(2):283-7.
21. Rotsaert A, Reyniers T, Jacobs BKM, Vanbaelen T, Burm C, Kenyon C, et al. PrEP user profiles, dynamics of PrEP use and follow-up: a cohort analysis at a Belgian HIV centre (2017-2020). Journal of the International AIDS Society. 2022;25(7):e25953.
22. Grinsztejn B, Hoagland B, Moreira RI, Kallas EG, Madruga JV, Goulart S, et al. Retention, engagement, and adherence to pre-exposure prophylaxis for men who have sex with men and transgender women in PrEP Brasil: 48 week results of a demonstration study. Lancet HIV. 2018;5(3):e136-e45.
23. Hosek SG, Rudy B, Landovitz R, Kapogiannis B, Siberry G, Rutledge B, et al. An HIV Preexposure Prophylaxis Demonstration Project and Safety Study for Young MSM. J Acquir Immune Defic Syndr. 2017;74(1):21-9.
24. Kwan TH, Lui GCY, Lam TTN, Lee KCK, Wong NS, Chan DPC, et al. Comparison between daily and on-demand PrEP (pre-exposure prophylaxis) regimen in covering condomless anal intercourse for men who have sex with men in Hong Kong: A randomized, controlled, open-label, crossover trial. Journal of the International AIDS Society. 2021;24(9):e25795.
25. Traikiatphum J, Wongharn P, Moonwong J, Songtaweesin WN, Vitsupakorn S, Premgamone A, et al. Retention in event-driven PrEP among young Thai men who have sex with men at risk of HIV acquisition. International journal of STD & AIDS. 2022;33(8):799-805.
26. Centers for Disease Control and Prevention. US Public Health Service: Preexposure prophylaxis for the prevention of HIV infection in the United States—2017 Update: a clinical practice guideline. Available from: https://www.cdc.gov/hiv/pdf/risk/prep/cdc-hiv-prep-guidelines-2017.pdf.
27. Chan PA, Patel RR, Mena L, Marshall BD, Rose J, Sutten Coats C, et al. Long-term retention in pre-exposure prophylaxis care among men who have sex with men and transgender women in the United States. Journal of the International AIDS Society. 2019;22(8):e25385.
28. General Statistics Office of Vietnam - Ministry of Planning and Investment. Announcement of population survey of life costing Hanoi2022 [Available from: https://www.gso.gov.vn/du-lieu-va-so-lieu-thong-ke/2022/06/thong-cao-bao-chi-ket-qua-khao-sat-muc-song-dan-cu-2021/.
29. The World Bank Group. Official exchange rate (LCU per US$, period average) 2022 [cited 2022 10 September]. Available from: https://data.worldbank.org/indicator/PA.NUS.FCRF.
30. World Bank. GDP per capita (current US$) 2023 [cited 2022 16 May]. Available from: https://data.worldbank.org/indicator/NY.GDP.PCAP.CD.

For peer review only - http://bmjopen.bmj.com/site/about/guidelines.xhtml

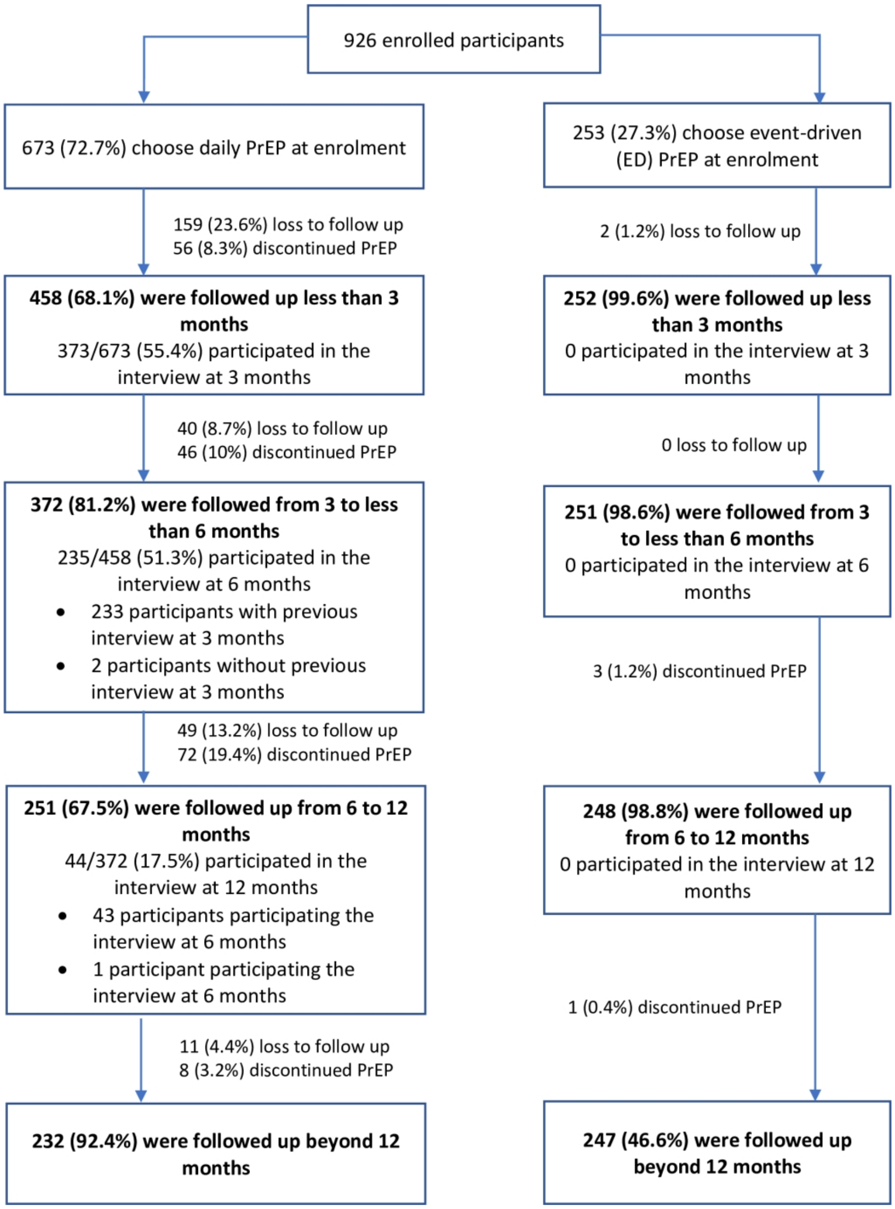

Figure 1. Flowchart of study participants.

159x214mm (300 x 300 DPI)

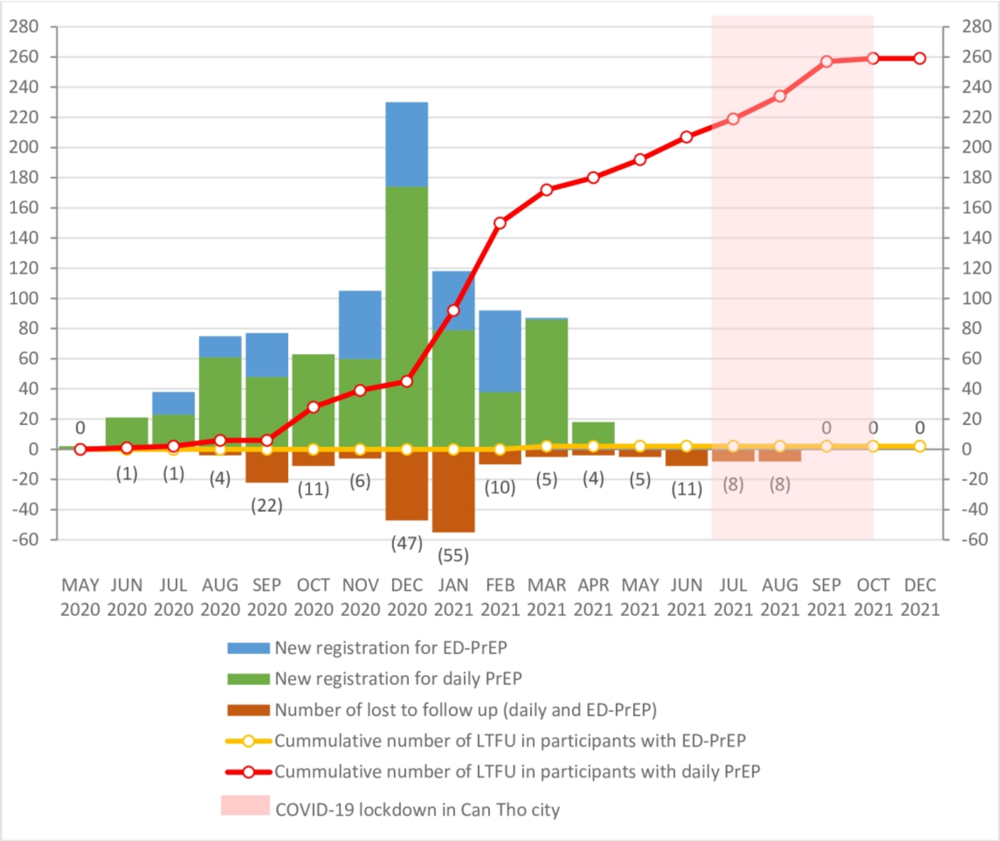

Figure 2. Number of new PrEP initiation and lost to follow up after the study enrolment

159x134mm (300 x 300 DPI)

Supplementary table 1. Baseline characteristic of participants using daily PrEP by the retention (excluding participants who decide to discontinue PrEP due to decreased HIV risk perception)

	All participants (n=491)	Retained in care (n=232)	Lost to follow up (n=259)	P value
**Sex assigned at birth**				
Male	465 (94.7%)	220 (94.8%)	245 (94.6%)	0.908
Female	26 (5.3%)	12 (5.2%)	14 (5.4%)	
**Sexual partners**				
No answer	1 (0.2%)	1 (0.4%)	0 (0.0%)	0.295
Men exclusively	419 (85.3%)	202 (87.1%)	217 (83.8%)	
Men and women	71 (14.5%)	29 (12.5%)	42 (16.2%)	
**HIV exposure within the past 3 days**				
No HIV exposure	488 (99.4%)	231 (99.6%)	257 (99.2%)	0.637
HIV exposure	2 (0.4%)	1 (0.4%)	1 (0.4%)	
No answer	1 (0.2%)	0 (0.0%)	1 (0.4%)	
**Frequency of sexual activity**				
≤ 2 times per week	169 (34.4%)	107 (46.1%)	62 (23.9%)	<0.001
>2 times per week	264 (53.8%)	105 (45.3%)	159 (61.4%)	
No answer	58 (11.8%)	20 (8.6%)	38 (14.7%)	
**Having sex without condom with people who were at risk of HIV within the past 6 months**				
No	130 (26.5%)	53 (22.8%)	77 (29.7%)	0.060
Yes	312 (63.5%)	160 (69.0%)	152 (58.7%)	
No answer	49 (10.0%)	19 (8.2%)	30 (11.6%)	
**Number of sexual partner within the past 6 months**				
1 sexual partner	115 (23.4%)	64 (27.6%)	51 (19.7%)	0.102
At least 2 sexual partners	370 (75.4%)	166 (71.6%)	204 (78.8%)	
No answer	6 (1.2%)	2 (0.9%)	4 (1.5%)	
**Having sex for money or gifts within the past 6 months**				
No	461 (93.9%)	225 (97.0%)	236 (91.1%)	0.017
Yes	21 (4.3%)	6 (2.6%)	15 (5.8%)	
No answer	9 (1.8%)	1 (0.4%)	8 (3.1%)	
**Diagnosis and/or treatment with an STI within the past 6 months**				
No	439 (89.4%)	208 (89.7%)	231 (89.2%)	0.006
Yes	39 (7.9%)	23 (9.9%)	16 (6.2%)	
No answer	13 (2.6%)	1 (0.4%)	12 (4.6%)	
**Sharing needles with other people within the past 6 months**				
No	486 (99.8%)	230 (100.0%)	256 (99.6%)	0.344

For peer review only

Yes	0 (0.0%)	0 (0.0%)	0 (0.0%)	
No answer	1 (0.2%)	0 (0.0%)	1 (0.4%)	
**Used PrEP within the past 6 months**				
No	451 (92.6%)	199 (86.5%)	252 (98.1%)	<0.0001
Yes	33 (6.8%)	31 (13.5%)	2 (0.8%)	
No answer	3 (0.6%)	0 (0.0%)	3 (1.2%)	
**Willingness to pay for PrEP**				
< $4.25/month	100 (20.4%)	44 (19.0%)	56 (21.6%)	0.101
$4.25-$14.89/month	302 (61.5%)	138 (59.5%)	164 (63.3%)	
$14.89-42.55$/month	67 (13.6%)	41 (17.7%)	26 (10.0%)	
>$42.55/month	1 (0.2%)	1 (0.4%)	0 (0.0%)	
Don’t want/unable to pay for PrEP	21 (4.3%)	8 (3.4%)	13 (5.0%)	

For peer review only

Supplementary table 3. Concerns about potential barriers to access PrEP at baseline

	All	Daily PrEP	ED-PrEP
Anticipated barrier to PrEP	338 (36.5%)	265 (39.4%)	73 (28.9%)
Concern about side effects	9 (1.0%)	9 (1.3%)	0 (0.0%)
Difficult in managing the time for visiting clinic and drug pick up PrEP	8 (0.9%)	5 (0.7%)	3 (1.2%)
Concerned about stigma/community perception	8 (0.9%)	8 (1.2%)	0 (0.0%)
Concerned about family finding out	6 (0.6%)	6 (0.9%)	0 (0.0%)
Concern about cost	6 (0.6%)	6 (0.9%)	0 (0.0%)
Concerned about stigma/community perception	4 (0.4%)	4 (0.6%)	0 (0.0%)
Difficulty in planning sex in advance	3 (0.3%)	1 (0.1%)	2 (0.8%)
Concerned about carrying the pills	2 (0.2%)	2 (0.3%)	0 (0.0%)
Difficulty in taking the pills 2 hours before sex	1 (0.1%)	1 (0.1%)	0 (0.0%)
Difficulty in remembering to take tablet everyday	1 (0.1%)	0 (0.0%)	1 (0.4%)
Drug storage	1 (0.1%)	1 (0.1%)	0 (0.0%)
Difficulty in managing the time for visiting clinic and drug pick up	1 (0.1%)	0 (0.0%)	1 (0.4%)
Concerned on the comorbidity of asthma	1 (0.1%)	0 (0.0%)	1 (0.4%)
Difficulty in transportation of PrEP	0 (0.0%)	0 (0.0%)	0 (0.0%)
Concerned about partner finding out	0 (0.0%)	0 (0.0%)	0 (0.0%)
Difficulty in remembering to take the following doses	0 (0.0%)	0 (0.0%)	0 (0.0%)
Difficulty in accessing STI, hepatitis testing and/or treatment	0 (0.0%)	0 (0.0%)	0 (0.0%)

Supplementary table 3. Challenges reported by daily PrEP users at 3, 6 and 12 months interviews

	3 months N= 373	6 months N=235	12 months N=44
**Willing to pay for PrEP per month**			

Don't want/unable to pay for PrEP	19 (5.1%)	9 (3.8%)	0 (0.0%)
>$42.55	1 (0.3%)	1 (0.4%)	0 (0.0%)
$14.89-42.55$	45 (12.1%)	36 (15.3%)	21 (47.7%)
$4.25-$14.89	240 (64.3%)	152 (64.7%)	22 (50.0%)
< $4.25	68 (18.2%)	36 (15.3%)	1 (2.3%)
**In the past 3 months, do you use daily PrEP or ED PrEP?**			
Both daily and ED PrEP	17 (4.6%)	3 (1.3%)	0 (0.0%)
Daily PrEP	296 (79.4%)	190 (80.9%)	36 (81.8%)
Any difficulties in taking PrEP medication in the past 3 months	110 (29.5%)	22 (9.4%)	13 (29.5%)
Forgot to take one or more PrEP doses	37 (9.9%)	9 (3.8%)	5 (11.4%)
Fitting PrEP into daily routine (for daily PrEP users)	7 (1.9%)	1 (0.4%)	1 (2.3%)
Unsure when to take PrEP medications	1 (0.3%)	1 (0.4%)	0 (0.0%)
Didn't know what to do about a missed dose	5 (1.3%)	3 (1.3%)	2 (4.5%)
Concerns about interactions with other medications	11 (2.9%)	5 (2.1%)	7 (15.9%)
Concerns about interactions with alcohol	2 (0.5%)	3 (1.3%)	2 (4.5%)
Concerns about interactions with hormonal therapy	1 (0.3%)	1 (0.4%)	0 (0.0%)
Have you had any difficulties accessing PrEP services?	131 (35.1%)	53 (22.6%)	18 (40.9%)
Difficult in managing the time for visiting clinic and drug pick up	37 (9.9%)	18 (7.7%)	6 (13.6%)
Transportation difficulty	11 (2.9%)	13 (5.5%)	13 (29.5%)
Concerned about partner finding out	5 (1.3%)	5 (2.1%)	1 (2.3%)
Concerned about family finding out	32 (8.6%)	12 (5.1%)	2 (4.5%)
Concerned about stigma/community perception	9 (2.4%)	5 (2.1%)	0 (0.0%)
Difficulty in remembering to take tablet everyday	17 (4.6%)	2 (0.9%)	0 (0.0%)
Carrying the pills with me	13 (3.5%)	7 (3.0%)	5 (11.4%)
Concern about side effects	52 (13.9%)	12 (5.1%)	4 (9.1%)
Concern about cost	9 (2.4%)	1 (0.4%)	0 (0.0%)
Difficulty in accessing STI, Hepatitis testing and/or treatment	5 (1.3%)	3 (1.3%)	1 (2.3%)
Health services closed due to Covid-19	33 (8.8%)	21 (8.9%)	16 (36.4%)

For peer review only

Not able to leave home due to Covid-19 lockdown	34 (9.1%)	27 (11.5%)	15 (34.1%)
**Side effects**			
Sleepy	1 (0.3%)	0 (0.0%)	0 (0.0%)
Nausea	12 (3.2%)	1 (0.4%)	1 (2.3%)
Muscle pain	1 (0.3%)	0 (0.0%)	0 (0.0%)
Loss of appetite	2 (0.5%)	0 (0.0%)	0 (0.0%)
Headache	3 (0.8%)	3 (1.3%)	2 (4.5%)
Dry lips	5 (1.3%)	2 (0.9%)	1 (2.3%)
Burning	5 (1.3%)	2 (0.9%)	4 (9.1%)
Acne	0 (0.0%)	0 (0.0%)	2 (4.5%)
Hyperpigmentation	0 (0.0%)	0 (0.0%)	0 (0.0%)
Fatigue	7 (1.9%)	6 (2.5%)	1 (2.3%)
Dizziness	7 (1.9%)	0 (0.0%)	0 (0.0%)
Dry skin	0 (0.0%)	0 (0.0%)	1 (2.3%)

Supplementary table 4. Number of participants switching between daily and ED-PrEP during the following up period

	Regimen	Participants preferred a daily PrEP regimen	
**During the first 3 months of PrEP use**	Daily PrEP	296	80.0%
	ED-PrEP	57	15.4%
	Switching between daily and ED-PrEP	17	4.6%
	Subtotal	370	100.0%
**Between 3 months and 6 months of PrEP use**	Daily PrEP	190	81.2%
	ED-PrEP	41	17.5%

For peer review only

	Switching between daily and ED-PrEP	3	1.3%
	Subtotal	234	100.0%
**Between 9 and 12 months of PrEP use**	Daily PrEP	36	83.7%
	ED-PrEP	7	16.3%
	Switching between daily and ED-PrEP	0	0.0%
	Subtotal	43	100.0%

For peer review only

STROBE Statement—checklist of items that should be included in reports of observational studies

	Item No	Recommendation	Page No
**Title and abstract**	1	(*a*) Indicate the study's design with a commonly used term in the title or the abstract	1
		(*b*) Provide in the abstract an informative and balanced summary of what was done and what was found	2
**Introduction**			
Background/rationale	2	Explain the scientific background and rationale for the investigation being reported	4
Objectives	3	State specific objectives, including any prespecified hypotheses	5
**Methods**			
Study design	4	Present key elements of study design early in the paper	5
Setting	5	Describe the setting, locations, and relevant dates, including periods of recruitment, exposure, follow-up, and data collection	5
Participants	6	(*a*) Give the eligibility criteria, and the sources and methods of selection of participants	5
Variables	7	Clearly define all outcomes, exposures, predictors, potential confounders, and effect modifiers. Give diagnostic criteria, if applicable	5
Data sources/ measurement	8*	For each variable of interest, give sources of data and details of methods of assessment (measurement). Describe comparability of assessment methods if there is more than one group	*6*
Bias	9	Describe any efforts to address potential sources of bias	9
Study size	10	Explain how the study size was arrived at	5
Quantitative variables	11	Explain how quantitative variables were handled in the analyses. If applicable, describe which groupings were chosen and why	NA
Statistical methods	12	(*a*) Describe all statistical methods, including those used to control for confounding	6
		(*b*) Describe any methods used to examine subgroups and interactions	6
		(*c*) Explain how missing data were addressed	Fig 11
		(*d*) If applicable, describe analytical methods taking account of sampling strategy	NA
		(*e*) Describe any sensitivity analyses	NA
**Results**			
Participants	13*	(a) Report numbers of individuals at each stage of study—eg numbers potentially eligible, examined for eligibility, confirmed eligible, included in the study, completing follow-up, and analysed	6
		(b) Give reasons for non-participation at each stage	6
		(c) Consider use of a flow diagram	
Descriptive data	14*	(a) Give characteristics of study participants (eg demographic, clinical, social) and information on exposures and potential confounders	6
		(b) Indicate number of participants with missing data for each variable of interest	6
Outcome data	15*	Report numbers of outcome events or summary measures	6

For peer review only - http://bmjopen.bmj.com/site/about/guidelines.xhtml

Main results	16	(*a*) Give unadjusted estimates and, if applicable, confounder-adjusted estimates and their precision (eg, 95% confidence interval). Make clear which confounders were adjusted for and why they were included	7
		(*b*) Report category boundaries when continuous variables were categorized	NA
		(*c*) If relevant, consider translating estimates of relative risk into absolute risk for a meaningful time period	NA
Other analyses	17	Report other analyses done—eg analyses of subgroups and interactions, and sensitivity analyses	7
**Discussion**			
Key results	18	Summarise key results with reference to study objectives	8
Limitations	19	Discuss limitations of the study, taking into account sources of potential bias or imprecision. Discuss both direction and magnitude of any potential bias	9
Interpretation	20	Give a cautious overall interpretation of results considering objectives, limitations, multiplicity of analyses, results from similar studies, and other relevant evidence	9
Generalisability	21	Discuss the generalisability (external validity) of the study results	9
**Other information**			
Funding	22	Give the source of funding and the role of the funders for the present study and, if applicable, for the original study on which the present article is based	8

*Give information separately for exposed and unexposed groups.

Note: An Explanation and Elaboration article discusses each checklist item and gives methodological background and published examples of transparent reporting. The STROBE checklist is best used in conjunction with this article (freely available on the Web sites of PLoS Medicine at http://www.plosmedicine.org/, Annals of Internal Medicine at http://www.annals.org/, and Epidemiology at http://www.epidem.com/). Information on the STROBE Initiative is available at www.strobe-statement.org.

For peer review only - http://bmjopen.bmj.com/site/about/guidelines.xhtml

BMJ Open

Preference and retention of daily and event-driven pre-exposure prophylaxis for HIV prevention: a prospective cohort in Can Tho city, Viet Nam

Journal:	*BMJ Open*
Manuscript ID	bmjopen-2023-075976.R2
Article Type:	Original research
Date Submitted by the Author:	10-Jan-2024
Complete List of Authors:	Nguyen, Van Thi Thuy; World Health Organization, WHO Viet Nam<br>Dat, Vu Quoc; Hanoi Medical University, Department of Infectious Diseases; Hanoi Medical University, Hanoi Medical University Hospital<br>Truc, Huynh Minh; City Center for Disease Control and Prevention<br>Nguyen Anh Thu, Pham; City Center for Disease Control and Prevention<br>Linh, Doan Thi Thuy; Viet Nam Administration for HIV/AIDS Control<br>Johnson, Cheryl; World Health Organization, Department of HIV/AIDS<br>Baggaley, R; World Health Organization<br>Phan, Huong; Viet Nam Administration for HIV/AIDS Control
**Primary Subject Heading**:	HIV/AIDS
Secondary Subject Heading:	Public health
Keywords:	HIV & AIDS < INFECTIOUS DISEASES, Health Services Accessibility, PREVENTIVE MEDICINE

SCHOLARONE™
Manuscripts

I, the Submitting Author has the right to grant and does grant on behalf of all authors of the Work (as defined in the below author licence), an exclusive licence and/or a non-exclusive licence for contributions from authors who are: i) UK Crown employees; ii) where BMJ has agreed a CC-BY licence shall apply, and/or iii) in accordance with the terms applicable for US Federal Government officers or employees acting as part of their official duties; on a worldwide, perpetual, irrevocable, royalty-free basis to BMJ Publishing Group Ltd ("BMJ") its licensees and where the relevant Journal is co-owned by BMJ to the co-owners of the Journal, to publish the Work in this journal and any other BMJ products and to exploit all rights, as set out in our licence.

The Submitting Author accepts and understands that any supply made under these terms is made by BMJ to the Submitting Author unless you are acting as an employee on behalf of your employer or a postgraduate student of an affiliated institution which is paying any applicable article publishing charge ("APC") for Open Access articles. Where the Submitting Author wishes to make the Work available on an Open Access basis (and intends to pay the relevant APC), the terms of reuse of such Open Access shall be governed by a Creative Commons licence – details of these licences and which Creative Commons licence will apply to this Work are set out in our licence referred to above.

Other than as permitted in any relevant BMJ Author's Self Archiving Policies, I confirm this Work has not been accepted for publication elsewhere, is not being considered for publication elsewhere and does not duplicate material already published. I confirm all authors consent to publication of this Work and authorise the granting of this licence.

For peer review only

Preference and retention of daily and event-driven pre-exposure prophylaxis for HIV prevention: a prospective cohort in Can Tho city, Viet Nam

Running Title: Pre-exposure prophylaxis for HIV in Viet Nam

Van Thi Thuy Nguyen[1¥], Vu Quoc Dat[2,3¥*], Huynh Minh Truc[4], Pham Nguyen Anh Thu[4], Doan Thi Thuy Linh[5], Cheryl Johnson[6], Rachel Baggaley[6], Phan Thi Thu Huong[5]

[1] World Health Organization - Viet Nam Country Office, Hanoi, Viet Nam

[2] Department of Infectious Diseases, Hanoi Medical University, Hanoi, Viet Nam

[3] Hanoi Medical University Hospital, Hanoi Medical University, Hanoi, Viet Nam

[4] City Center for Disease Control and Prevention, Can Tho city, Viet Nam

[5] Viet Nam Administration for HIV/AIDS Control, Viet Nam

[6] World Health Organization, Global HIV, Viral hepatitis and STIs programmes, Geneva, Switzerland

[¥] Co-first author

*Corresponding author: Vu Quoc Dat, MD, PhD. Email: datvq@hmu.edu.vn. Postal address: No 1 Ton That Tung Street, Hanoi, Viet Nam.

For peer review only - http://bmjopen.bmj.com/site/about/guidelines.xhtml

Abstract

Objective

Pre-exposure prophylaxis (PrEP) was introduced in Viet Nam in 2017, but data on oral PrEP preference and effective use beyond 3 months are limited. We aimed to evaluate PrEP preferences for PrEP, factors influencing uptake, choice and effective use, as well as barriers to PrEP.

Methods

This is a prospective cohort study in Can Tho, Viet Nam. Participants who were eligible for PrEP and provided informed consent were interviewed at baseline on demographic information, willingness to pay, reasons for choosing their PrEP regimen and the anticipated difficulties in taking PrEP and followed-up at 3 months, 6 months and 12 months after PrEP initiation.

Findings

Between May 2020 and April 2021, 926 individuals at substantial risk for HIV initiated PrEP. Of whom 673 (72.7%) choose daily PrEP and 253 (27.3%) choose event-driven (ED)-PrEP. The majority of participants were men (92.7%) and only 6.8% were women and 0.5% were transgender women. Median participant age was 24 years (IQR 20-28) and 84.7% reported as exclusively same sex-relationship. The three most common reasons for choosing daily PrEP were effectiveness (24.3%) and unplanning for sex (22.9%). Those opting for ED-PrEP also cited effectiveness (22.7%), as well as convenience (18.0%) and easier effective-use 12.0%). Only 7.8% of PrEP users indicated they were unwilling to pay for PrEP and 76.4% would be willing to pay if PrEP were less than US $15 per month. The proportion of user effectively using PrEP at 12 months was 43.1% and 99.2% in daily PrEP and ED-PrEP users, respectively.

Conclusions

Event-driven PrEP was preferred by more than a quarter of 23.5% of the participants and there was little concern about potential adverse events. High rates of effective use were reported by ED-PrEP users. Future research to inform implementation of PrEP in Viet Nam is needed to develop ways of measuring adherence to ED-PrEP more accurately and to understand and address difficulties in taking daily PrEP use.

Keywords: PrEP, pre-exposure prophylaxis, daily PrEP, event-driven PrEP, on-demand PrEP, MSM, retention, Viet Nam

For peer review only - http://bmjopen.bmj.com/site/about/guidelines.xhtml

For peer review only

Strengths and limitations of this study

- We conducted the first study on preference, retention and factors associated with these in PrEP use in Viet Nam.
- The major limitations related to the study design of single center
- The self-statement of adherence among ED-PrEP users in this study could contribute to overestimate of the retention.

For peer review only

For peer review only - http://bmjopen.bmj.com/site/about/guidelines.xhtml

Introduction

As of December 2020, Viet Nam reported that there were 215,220 people with HIV, in which there were 12,200 new HIV infections and 1,681 AIDS-related deaths in 2020 (1). The HIV epidemic in Viet Nam is concentrated in key populations including people who inject drugs (PWID), men who have sex with men (MSM) and female sex workers (FSWs). It is estimated that there are approximately 200,000 MSM in Viet Nam(2). In recent years, HIV prevalence has increased in the MSM population, from 5.1% in 2015 to 13.3% in 2020, while prevalence was stable in PWID populations (12.7% in 2019) and FSWs (3.1% in 2020) (1).

Since 2016, the World Health Organization (WHO) has recommended oral pre-exposure prophylaxis (PrEP) to further reduce new infections among populations where HIV incidence and risk is high. Following this guidance, between June and December 2017, Viet Nam updated their national guidelines and started initial PrEP implementation in Hanoi and Ho Chi Minh city. Since then, PrEP implementation in Viet Nam has continued to expand and as of August of 2021, there were nearly 32,000 persons using PrEP across 200 clinics in nearly half of all provinces in the country(3). Current national guidelines recommend daily-PrEP (tenofovir disoproxil fumarate (TDF) co-formulated with emtricitabine (FTC) or lamivudine (3TC)) for populations at substantial risk and event-driven PrEP (ED-PrEP) (TDF/XTC) for MSM who have less frequent sex (<2 times per week) (4).

While oral PrEP continues to expand and be an effective option for many, recent evidence has highlighted that more differentiated service delivery options are needed. In particular, ED-PrEP provides an effective option which removes the need for daily doses and for use before and after high risk sex. Among MSM, ED-PrEP has been shown to reduce HIV transmission by up to 86% (5). Studies have also shown that MSM may often prefer ED-PrEP over daily oral PrEP because of its convenience. In a US-survey, 74.3% of MSM who were hesitant to start oral daily PrEP indicated that they would be more willing to try oral ED-PrEP (6). In Thailand some PrEP users considered daily regimens the easiest to use, as it could be incorporated into daily routines and did not require planning for sex. These men expressed concerns, however, about the long-term safety and affordability of daily oral dosing (7). Study participants appreciated oral ED-PrEP for minimizing drug exposure and potential adverse events. They considered ED-PrEP an attractive choice for MSM who had infrequent sex, were able to plan for sex, and had the ability to take the post-sex dose (7).

Despite the potential benefits of ED-PrEP, it is little known about preference and uptake of ED-PrEP among MSM in Viet Nam. Thus, this study aims to assess both preferences as well as actual uptake and continuation of oral daily and ED-PrEP among MSM in Viet Nam to inform future

For peer review only - http://bmjopen.bmj.com/site/about/guidelines.xhtml

programming. In addition, difficulties related to PrEP uptake and continuation including COVID-19-related issues were explored to inform future differentiated PrEP service delivery models.

Methods

Study design and participants

We conducted a prospective study in all 11 PrEP clinics in Can Tho which has the highest HIV prevalence among MSM (22.7%)(2). MSM were referred to the PrEP clinics from community-based HIV testing led by MSM groups or via self-referral. All clinics were integrated with HIV testing and/or ART services. PrEP eligibility was evaluated following the national guideline: (1) confirmed HIV-negative status, (2) no signs and symptoms of acute HIV infection and (3) at substantial risk for HIV infection within past 6 months. We defined substantial risk as any of the following: individual engaged in condomless anal or vaginal sex, having at least 2 sexual partners, reported sexual partner with substantial risk for HIV infection, or having a sexual partner with HIV but not currently on ART or with unknown/detectable viral load (>200 copies/ml), who had been previously diagnosed with a sexually transmitted infection (STI), and who reported having multiple courses of PEP and continued sexual risk behaviour. Only eligible participants aged 16 years and over who agreed to participate and provide written informed consent were recruited for the study.

Study procedure and data collection

In the community-based setting, PrEP screening and offering different PrEP regimen is part of HIV post-test counselling (HTC) (8). Clients who were interested in PrEP will be referred to a PrEP clinic. At the PrEP clinics, clients were evaluated based on their behavioural risk to assess PrEP eligibility. ED-PrEP were offered for MSM who have infrequent sex ($\leq$ 2 times per week on average) and are usually able to plan for sex at least two hours in advance, or who can delay sex for at least two hours or their own preference of ED-PrEP. During screening, the clinic staff explained what PrEP is, the benefits and differences between daily PrEP and ED-PrEP and let the client decide. After the clients chose their preferred PrEP regimen, they were invited to provide informed consent and participate in the study and provide written informed consents. Daily PrEP regimens were offered based on the availability of the antiretroviral including tenofovir disoproxil fumarate/emtricitabine (TDF/FTC) or tenofovir disoproxil fumarate/lamivudine (TDF/3TC) or TDF. ED- PrEP regimens were offered as TDF/FTC or TDF/3TC

National guidelines recommend follow-up with all PrEP clients at health facilities starting with 1-2 months after PrEP initiation and then quarterly thereafter. We used a questionnaire consisting of six questions on willingness to pay (closed-end questions. Willingness to pay estimates were reported in 2,000 Vietnam Dong (VND) during the interview and converted to US dollar (2021) for this analysis

For peer review only - http://bmjopen.bmj.com/site/about/guidelines.xhtml

(1 US$ = 23,529 Viet Nam Dong). Semi-structured interviews were used to understand potential barriers to PrEP use. PrEP users were monitored following Viet Nam Ministry of Health's guidelines including HIV testing and continuation. Continuation of PrEP was defined if PrEP users who come back to pick up drugs (for daily PrEP) or self-reported to adherence (for ED-PrEP) at the corresponding following up visits after initiation at 3-, 6- and 12-month visits.

Data Analysis

Participants' responses to the open-ended questions were coded by an independent investigator (VQD). Statistical analysis was performed using the IBM SPSS Statistics for Windows, Version 27.0 (Armonk, NY: IBM Corp). We used conventional descriptive statistics to summarise the characteristics of the study's participants and their views on PrEP. Effective use was calculated by dividing the number of PrEP users retained at 3, 6 and 12 months of PrEP by the total number of clients enrolled in PrEP study and multiplying by 100. Multivariable logistic regression was used to estimate adjusted odd ratio (aOR) and 95% CIs for PrEP retention by selected baseline characteristics. P-value < 0.05 were considered statistically significant.

Ethics

The study was approved by Hanoi Medical University Institutional Ethical Review Board (IRB VN01.001/IRB 0000312/FWA 00004148), and WHO Wester Pacific Regional Office Ethical Review Committee (2020.4.VTN.1.HSI).

Patient and public involvement

No patient or public involvement.

Results

Between May 2020 and Apr 2021, we enrolled 926 clients of whom 253 (27.3%) choose ED-PrEP and 673 (72.7%) choose daily PrEP at enrolment. Participants were follow-up until 31 December 2022. Table 1 and Supplementary table 1 show the characteristics of enrolled participants. The median age was 24 years (IQR 20-28) and ranging from 16 to 51 years. Twelve participants were under 18 years old (1.3%). The majority of men participants reported their exclusive sex with men (784/926 or 84.7%) and there was no significant difference in age and gender identity between groups of daily PrEP and ED-PrEP.

At baseline, 94.1% (871/926) participants responded to an open-ended question on the reasons that determined their PrEP preference after the consultation with the clinic staff. The response rate for reporting the reasons PrEP preference was not significant between the groups of daily PrEP (94.8%; 638/673) and ED-PrEP (233/253 or 91.1%). The most common reasons for choosing daily-PrEP were

For peer review only - http://bmjopen.bmj.com/site/about/guidelines.xhtml

For peer review only

the effectiveness of PrEP in preventing HIV infection (155/638 or 24.3%), preventing the transmission of HIV following condomless sex (146/638 or 22.9%) and easy for longer-term use (121/638 or 19.0%). Among participants who chose ED-PrEP, the most common reasons were effectiveness (53/233 or 22.7%), convenience (42/233 or 18%) and easy for adherence (28/233 or 12.0%). The detailed reasons for PrEP preference at baseline was showed in the Table 2. Among 338/926 (36.5%) participants who anticipated PrEP barriers, only 22/338 (6.5%) (17/265 preferred daily PrEP and 5/73 preferred ED-PrEP) expressed the specific concerns on PrEP (Supplementary table 2).

Regarding the willingness to pay for PrEP, most (76.4%) were willing to pay for PrEP if less than US $15 per month, while some (7.8%) said they would not pay or felt they were unable to pay for PrEP at any cost. The proportions of clients willing to pay at different prices were statistically different between daily and ED-PrEP groups (Table 1).

The median follow-up time was 284 days (IQR 102–367) among 926 participants who initiated PrEP, 214 days (IQR 60-323) in participants choosing daily PrEP and 363 days (IQR 319-389) in participants choosing ED-PrEP. By the end of the study, 261/926 (28.2%) patients were lost to follow- up, 186/926 (20.1%) discontinued and 479/926 (51.7%) were on PrEP (Figure 1). Much of the loss to follow-up occurred within the first 3 months of enrolment (159/261 or 60.9%) and among those taking daily oral PrEP (259/261 or 99.2%). The overall retention rates at 3, 6 and 12 months in the daily PrEP group were 72.6% (439/605), 64.5% (363/563) and 43.1% (150/198), respectively with the median time of lost to follow-up of 60 days. The retention rates in the ED-PrEP group were 99.2% (251/253) at 3 and 6 months and 99.4% (158/159) at 12 months. Of 186 participants who discontinued PrEP, reasons reported for discontinuation were that they were no longer sexually active (87/186 or 46.8%), moving to a new place (85/186 or 45.7%), were diagnosed with HIV (seroconversion) (7/186 or 3.8%), had different user preferences, had concerns about medication related toxicities, were diagnosed with HBV (each of 2/185 or 1.1%) or were affected due to COVID-19-related restrictions (1/186 or 0.5%).

Of participants who started daily PrEP and completed the interview, the proportions of participants reporting any PrEP side effects were 32/341 (8.6%) at 3 months, 12/235 (5.1%) at 6 months and 5/44 (11.4%) at 12 months. The detail of reported side effects was listed in the Supplementary table 3.

During COVID-19 pandemic, Can Tho city was locked down due to COVID-19 outbreak (e.g from July – October 2021). However, as shown in the Figure 2, a larger percentage of participants discontinued PrEP before the lockdown period. There were only 40/261 (15.3%) participants who lost to follow up within 4 months of lockdown and before of the study completion.

For peer review only - http://bmjopen.bmj.com/site/about/guidelines.xhtml

We also carried out multivariable logistic regression to determine factors associated with PrEP retention among daily PrEP participants (continued daily PrEP group vs lost-to-follow-up group). The results indicate that factors associated with daily PrEP continuation were less frequency of sex (less than twice per week) (aOR 2.199, 95%CI 1.306-3.702,), having sex without condom with people who were at risk of HIV within the past 6 months (aOR 1.991, 95%CI 1.180-3.362), PrEP use within the past 6 mnths (aOR 13.568, 95%CI 3.015-61.071) and no anticipation of barriers to PrEP use (aOR 1.721, 95%CI 1.042-2.842) (Table 3). Due to small number of lost to follow-up among ED-PrEP users, we were unable to execute multivariable Cox regression for this group.

Discussion

This study is the first prospective study in Viet Nam to explore both PrEP preferences and use, as well as effective use and factors associated with PrEP continuation. We found that among individuals who were eligible for PrEP, more than a quarter preferred ED-PrEP over daily PrEP. The proportion of the MSM who preferred ED-PrEP in our study was similar to the studies in high income countries, including Belgium (23.4%-23.5%) (9, 10), the Netherland (26.7%-27.3%)(10, 11) and Australia (~20%)(12, 13) but was lower than countries such as France (49.5%)(14), Taiwan (56%)(15), China (57.1%)(16) and others in West Africa (72.1%-74%)(17, 18). We reported a great variety of individual factors determine the choices for their PrEP regimens, mostly related to the participants' perceptions of PrEP efficacy in prevention of HIV transmission, safety, perceived adherence and convenience. In a qualitative study in 857 MSM on daily PrEP and 301 MSM on ED-PrEP in Netherlands, preference of oral PrEP was reported to include frequency of sex, expected adherence, perceived safety, efficacy and burden of the pills and anticipated side effects(19). In a prospective study in 1000 MSM who use oral PrEP in China, the multivariable marginal effect analysis show that factors associated with an increased preference for daily versus ED-PrEP were currently being married to or living with a female (adjusted marginal effect = −0.146 [95% CI: −0.230, −0.062], *p* = 0.001), number of male sexual partners in the previous six months (adjusted marginal effect = 0.003, 95% CI: 0.000- 0.005], *p* = 0.034) and a subjective assessment of being very high risk of HIV infection (adjusted marginal effect size = 0.105 [95% CI: 0.012, 0.198], *p* = 0.027)(16).

For PrEP effective use, we found that those on ED-PrEP had greater continuation rate compared to those opting for daily oral PrEP at 12 months (99.2% and 43.1%, p<0.001 respectively). Daily PrEP retention has been reported in different studies with large variations by study design, PrEP delivery approaches and countries. The proportion of retention at 12 months ranged from 43% in a study of 5,583 MSM from 2012 to 2017 in 6 clinical sites in United State (20), 72.3% in a cohort of 1347 PrEP users in Belgium between 2017 and 2020(21), 83% in 450 MSM in Brazil between 2014 and

For peer review only - http://bmjopen.bmj.com/site/about/guidelines.xhtml

2016(22) to 91.8% in a study of 400 MSM in 12 urban US cities in 2013 (23). In a randomized control trial with 119 MSM in Hong Kong, the oral daily and ED PrEP retention at 32 week was 86% and 87% respectively(24). Proportion of retention to ED-PrEP among Thai MSM aged 15-19 years was 88.9%, 95% CI: 73.9-96.9%) at 6 months(25). The definition of retention for PrEP was varied and made it difficult to compare the proportion of retention across the studies. It was conventionally defined as the return for follow-up every 3 months(26) or attendance at a specific timepoint (eg. 3, 6 and 12 months) with a time window (±30 days) while clients may not attend a follow up visit with a precise intervals(27), especially in case of ED-PrEP. In addition, concepts of retention are changing and that people come during periods of risk and have different needs. Thus, effective use of PrEP is increasingly being used while adherence and retention are not. Our findings suggest more diverse and flexible PrEP models might lead to better use and engagement from clients. These results can also be leveraged to improve oral daily PrEP by making services and follow-up more differentiated including use of HIVST use for PrEP continuation. We found that the majority of MSM in Can Tho were willing to pay for PrEP (92.2%). However, 82.8% (707/854) participants in our study indicated that their willingness to pay was low (<15 US$/month) (the average income per person in Mekong delta was 3,713,000 VND(28) or approximately US160.3, at the exchange rate US$1=23,159.8 VND in 2021(29)) while 65% of respondents in Thailand willing to pay US$25 (monthly average income per person was US$478.1 in 2012(30)) and 88.9% of respondents in China would like to pay > US$14 per month for PrEP (monthly average income per person was US$ 867.3 in 2020(30)).

We noticed that the number of new registration for PrEP and the number of lost-to-follow up were highest between December 2021 and January 2022. In Viet Nam, December was designated for the HIV action month with many events promoted for HIV interventions including PrEP, which may have been related to the increase in the new registration and also high rate of lost-to-follow up thereafter.

Limitation

Our limitation in this study was the patients were recruited from a single province; thus, the study site is purposely selected and may not represent all PrEP users in Viet Nam. The self-report on challenges during PrEP taking could be recall bias. In addition, we didn't included information on re-starting PrEP or switching from daily PrEP to ED-PrEP in this analysis which may cause a potential bias of underestimate the retention rate. Also, self-statement of adherence among ED-PrEP users could contribute to overestimate of the retention in this group.

For peer review only - http://bmjopen.bmj.com/site/about/guidelines.xhtml

Conclusion

Individuals at substantial risk for HIV especially MSM in Can Tho, Viet Nam were motivated to choose PrEP by their beliefs about the safety, efficacy and, frequency of sex and expected adherence with little concerns about side effects and specific barriers to use PrEP. ED-PrEP was desirable and achieved high levels of effective use in this cohort study, but with low willingness to pay. ED-PrEP is desirable and should be offered as an option to expand access and prevent new infections in Viet Nam. Further research is needed to provide more insights, particularly on loss to follow up and implementation of more flexible PrEP delivery models.

Data availability statement

Data are available upon reasonable request.

Funding

The study was funded by Viet Nam - WHO Country Office. Grant number: NA,

Contributors

VNTT, PTTH conceived and designed the study. VQD analysed the data and wrote the first draft of the report. VTTN, PTTH, HMT, PNAT, and DTTL involved to the acquisition and interpretation of data. VTTN, CJ, RB, SD and PTTH reviewed the manuscript and provided substantial contribution. All authors contributed to the manuscript finalization and approved the final report.

Competing interests

We declare no competing interest.

Acknowledgments

We are very grateful to the health staff at Can Tho CDC and the participating PrEP clinics in Can Tho who contributed to the implementation of the study.

For peer review only - http://bmjopen.bmj.com/site/about/guidelines.xhtml

Table 1. Characteristic of participants by the initial PrEP preference

Characteristics	All participants (n=926)	Participants preferred a daily PrEP regimen (n=673)	Participants preferred ED PrEP regimen (n=253)	p value
**Gender identity**				<0.001
Male	858 (92.7%)	608 (90.3%)	250 (98.8%)	
Female	63 (6.8%)	61 (9.1%)	2 (0.8%)	
Trans female	5 (0.5%)	4 (0.6%)	1 (0.4%)	
**Age (median, IQR) (years)**	24 (20-28)	24 (20-28)	23 (21-27)	
**Sexual partners**				0.568
No answer	3 (0.3%)	3 (0.4%)	0 (0.0%)	
Men exclusively	784 (84.7%)	569 (84.5%)	215 (85.0%)	
Men and women	139 (15%)	101 (15%)	38 (15%)	
**HIV exposure within the past 3 days**				0.224
No HIV exposure	915 (98.8%)	667 (99.1%)	248 (98.0%)	
HIV exposure	10 (1.1%)	5 (0.7%)	5 (2.0%)	
No answer	1 (0.1%)	1 (0.1%)	0 (0.0%)	
**Frequency of sexual activity**				<0.001
≤ 2 times per week	279 (32.1%)	228 (37.0%)	51 (20.2%)	
>2 times per week	561 (64.6%)	366 (59.4%)	195 (77.4%)	
No answer	28 (3.2%)	22 (3.6%)	6 (2.4%)	
**Having sex without condom with people who were at risk of HIV within the past 6 months**				<0.001
No	352 (38.0%)	183 (27.2%)	169 (66.8%)	
Yes	502 (54.2%)	424 (63.0%)	78 (30.8%)	
No answer	72 (7.8%)	66 (9.8%)	6 (2.4%)	
**Number of sexual partners within the past 6 months**				<0.001
1 sexual partner	205 (22.1%)	171 (25.4%)	36 (14.2%)	
At least 2 sexual partners	703 (75.9%)	495 (73.6%)	208 (82.2%)	

For peer review only - http://bmjopen.bmj.com/site/about/guidelines.xhtml

No answer	16 (1.7%)	7 (1.0%)	9 (3.6%)	
**Having sex for money or gifts within the past 6 months**				0.002
No	835 (90.2%)	619 (92.0%)	216 (85.4%)	
Yes	60 (6.5%)	32 (4.8%)	28 (11.1%)	
No answer	31 (3.3%)	22 (3.3%)	9 (3.6%)	
**Diagnosis and/or treatment with an STI within the past 6 months**				<0.001
No	793 (85.6%)	597 (88.7%)	196 (77.5%)	
Yes	103 (11.1%)	51 (7.6%)	52 (20.6%)	
No answer	30 (3.2%)	25 (3.7%)	5 (2.0%)	
**Sharing needles with other people within the past 6 months**				0.387
No	917 (99.5%)	664 (99.3%)	253 (100.0%)	
Yes	2 (0.2%)	2 (0.3%)	0 (0.0%)	
No answer	3 (0.3%)	3 (0.4%)	0 (0.0%)	
**Used PrEP within the past 6 months**				0.001
No	874 (94.8%)	623 (93.1%)	251 (99.2%)	
Yes	44 (4.8%)	42 (6.3%)	2 (0.8%)	
No answer	4 (0.4%)	4 (0.6%)	0 (0.0%)	
**Willingness to pay for PrEP**				<0.001
< US$4.25/month	196 (21.2%)	130 (19.3%)	66 (26.1%)	
US$4.25-US$14.89/month	511 (55.2%)	407 (60.5%)	104 (41.1%)	
US$14.89-US$42.55/month	145 (15.7%)	91 (13.5%)	54 (21.3%)	
>US$42.55/month	2 (0.2%)	2 (0.3%)	0 (0.0%)	
Don't want/unable to pay for PrEP	72 (7.8%)	43 (6.4%)	29 (11.5%)	

For peer review only - http://bmjopen.bmj.com/site/about/guidelines.xhtml

Figure 1. Flowchart of study participants.

For peer review only

For peer review only - http://bmjopen.bmj.com/site/about/guidelines.xhtml

Table 2. Reasons for choosing PrEP at baseline

Reasons	All participants	Daily PrEP	ED-PrEP
**Number of sex partners**			
Few sex partners	25 (2.9%)	25 (3.9%)	0 (0.0%)
Multiple sex partners	72 (8.3%)	71 (11.1%)	1 (0.4%)
**Frequency of sex**			
Having less frequent sex	19 (2.2%)	7 (1.1%)	12 (5.2%)
Having more frequent sex	104 (11.9%)	96 (15.0%)	8 (3.4%)
Effectively prevents HIV	208 (23.9%)	155 (24.3%)	53 (22.7%)
Prevents HIV following condomless sex	155 (17.8%)	146 (22.9%)	9 (3.9%)
Easy to adhere	149 (17.1%)	121 (19.0%)	28 (12.0%)
Convenient	79 (9.1%)	37 (5.8%)	42 (18.0%)
Helps protect others and oneself from HIV	60 (6.9%)	38 (6.0%)	22 (9.4%)
Safe (no concerns about side-effects)	52 (6.0%)	37 (5.8%)	15 (6.4%)
Marriage and/or staying with spouse/partner	33 (3.8%)	33 (5.2%)	0 (0.0%)
Having HIV positive sexual partner	29 (3.3%)	28 (4.4%)	1 (0.4%)
Wants to try something new	27 (3.1%)	1 (0.2%)	26 (11.2%)
Desires to feel safe (protected from HIV)	20 (2.3%)	20 (3.1%)	0 (0.0%)
Protection during vaginal sex	18 (2.1%)	18 (2.8%)	0 (0.0%)
Did not want to use condom	17 (2.0%)	8 (1.3%)	9 (3.9%)
PrEP option was free of charge	16 (1.8%)	4 (0.6%)	12 (5.2%)
Fears about broken condoms	10 (1.1%)	0 (0.0%)	10 (4.3%)
General fears about HIV	7 (0.8%)	2 (0.3%)	5 (2.1%)
Partner takes daily PrEP	3 (0.3%)	1 (0.2%)	2 (0.9%)
Protects one's family	2 (0.2%)	2 (0.3%)	0 (0.0%)
Doubts about their partner's fidelity	2 (0.2%)	1 (0.2%)	1 (0.4%)
Protection during oral sex	1 (0.1%)	1 (0.2%)	0 (0.0%)
Concerns about exposure to blood	1 (0.1%)	1 (0.2%)	0 (0.0%)

For peer review only

For peer review only - http://bmjopen.bmj.com/site/about/guidelines.xhtml

Figure 2. Number of new PrEP initiation and lost to follow up after the study enrolment

For peer review only

Table 3. Multivariate logistic regression predicting the retention in daily PrEP in Can Tho (*n* = 414)

Variables	Adjusted odd ratio (95%CI)	P value
**Age (1-yr. increment)**	0.967 (0.934-1.002)	0.065
**Gender identity**		
Male	1	
Female/trans female	1.932 (0.896-4.170)	0.093
**Sexual partners**		
Men and women	1	
Men exclusively	1.720 (0.919-3.218)	0.090
**Frequency of sexual activity**		
>2 times per week	1	
≤ 2 times per week	2.199 (1.306-3.702)	**0.003**
No answer	2.130 (0.927-4.897)	0.075
**Having sex without condom with people who were at risk of HIV within the past 6 months**		
No	1	
Yes	1.991 (1.180-3.362)	**0.010**
**Number of sexual partners within the past 6 months**		
≥2 partners	1	
<2 partners	1.225 (0.722-2.077)	0.452
**Having sex for money or gifts within the past 6 months**		
Yes	1	
No	3.259 (0.742-14.318)	0.118
**Diagnosis and/or treatment with an STI within the past 6 months**		
No	1	
Yes	0.795 (0.325-1.944)	0.615
**Used PrEP within the past 6 months**		
No	1	
Yes	13.568 (3.015-61.071)	**0.001**
**Willingness to pay for PrEP**		
Don't want/unable to pay for PrEP	1	
Willing to pay	0.832 (0.275-2.517)	0.744
**Anticipated barrier to PrEP (1)**		
No	1	
Yes	1.721 (1.042-2.842)	**0.034**

For peer review only - http://bmjopen.bmj.com/site/about/guidelines.xhtml

REFERENCES

1. Ministry of Health. Report on HIV/AIDS prevention in 2020. Hanoi; 2021 4 Feb. Report No.: 124/BC-BYT.
2. The Joint United Nations Programme on HIV and AIDS (UNAIDS), Vietnam Administration of AIDS Control (VAAC). A review of HIV prevention in Vietnam 2019. 2019.
3. Vietnam Administration of AIDS Control (VAAC). Interim guidance on implementation of PrEP in students. 2022.
4. Ministry of Health. Guidelines for the care and treatment of people living with HIV/AIDS 2021.
5. Molina JM, Charreau I, Spire B, Cotte L, Chas J, Capitant C, et al. Efficacy, safety, and effect on sexual behaviour of on-demand pre-exposure prophylaxis for HIV in men who have sex with men: an observational cohort study. Lancet HIV. 2017;4(9):e402-e10.
6. Beymer MR, Gildner JL, Holloway IW, Landovitz RJ. Acceptability of Injectable and On-Demand Pre-Exposure Prophylaxis Among an Online Sample of Young Men Who Have Sex with Men in California. LGBT Health. 2018;5(6):341-9.
7. Chemnasiri T, Varangrat A, Amico KR, Chitwarakorn A, Dye BJ, Grant RM, et al. Facilitators and barriers affecting PrEP adherence among Thai men who have sex with men (MSM) in the HPTN 067/ADAPT Study. AIDS Care. 2020;32(2):249-54.
8. Viet Nam Authority for HIV/AIDS Control. Guidance for implemenmentation of pre-exposure prophylaxis (PrEP). Hanoi: Viet Nam Authority for HIV/AIDS Control,; 2020.
9. Reyniers T, Nostlinger C, Laga M, De Baetselier I, Crucitti T, Wouters K, et al. Choosing Between Daily and Event-Driven Pre-exposure Prophylaxis: Results of a Belgian PrEP Demonstration Project. J Acquir Immune Defic Syndr. 2018;79(2):186-94.
10. Jongen VW, Reyniers T, Ypma ZM, Schim van der Loeff MF, Davidovich U, Zimmermann HM, et al. Choosing event-driven and daily HIV pre-exposure prophylaxis - data from two European PrEP demonstration projects among men who have sex with men. Journal of the International AIDS Society. 2021;24(8):e25768.
11. Coyer L, van den Elshout MAM, Achterbergh RCA, Matser A, Schim van der Loeff MF, Davidovich U, et al. Understanding pre-exposure prophylaxis (PrEP) regimen use: Switching and discontinuing daily and event-driven PrEP among men who have sex with men. EClinicalMedicine. 2020;29-30:100650.
12. Vaccher SJ, Gianacas C, Templeton DJ, Poynten IM, Haire BG, Ooi C, et al. Baseline Preferences for Daily, Event-Driven, or Periodic HIV Pre-Exposure Prophylaxis among Gay and Bisexual Men in the PRELUDE Demonstration Project. Front Public Health. 2017;5:341.
13. C. Chan MH, T.R. Broady, J. MacGibbon, L. Mao,, G. Prestage BW, B. Clifton, B.R. Bavinton. Increased use of event-driven PrEP during COVID-19 in Australia: results from behavioural surveillance 2019-2021. The 12th IAS Conference on HIV Science (IAS 2023); Munich, Germany2023.
14. Molina JM, Ghosn J, Assoumou L, Delaugerre C, Algarte-Genin M, Pialoux G, et al. Daily and on-demand HIV pre-exposure prophylaxis with emtricitabine and tenofovir disoproxil (ANRS PREVENIR): a prospective observational cohort study. Lancet HIV. 2022;9(8):e554-e62.
15. Wu HJ, Wen-Wei Ku S, Chang HH, Li CW, Ko NY, Strong C. Imperfect adherence in real life: a prevention-effective perspective on adherence to daily and event-driven HIV pre-exposure prophylaxis among men who have sex with men - a prospective cohort study in Taiwan. Journal of the International AIDS Society. 2021;24(5):e25733.
16. Zhang J, Xu JJ, Wang HY, Huang XJ, Chen YK, Wang H, et al. Preference for daily versus on-demand pre-exposure prophylaxis for HIV and correlates among men who have sex with men: the China Real-world Oral PrEP Demonstration study. Journal of the International AIDS Society. 2021;24(2):e25667.

For peer review only - http://bmjopen.bmj.com/site/about/guidelines.xhtml

17. Laurent C, Dembele Keita B, Yaya I, Le Guicher G, Sagaon-Teyssier L, Agboyibor MK, et al. HIV pre-exposure prophylaxis for men who have sex with men in west Africa: a multicountry demonstration study. Lancet HIV. 2021;8(7):e420-e8.
18. Laurent C, Yaya I, Cuer B, Sagaon-Teyssier L, Mensah E, Dah TTE, et al. Human Immunodeficiency Virus Seroconversion Among Men Who Have Sex With Men Who Use Event-Driven or Daily Oral Pre-Exposure Prophylaxis (CohMSM-PrEP): A Multi-Country Demonstration Study From West Africa. Clin Infect Dis. 2023;77(4):606-14.
19. Zimmermann HM, Eekman SW, Achterbergh RC, Schim van der Loeff MF, Prins M, de Vries HJ, et al. Motives for choosing, switching and stopping daily or event-driven pre-exposure prophylaxis - a qualitative analysis. Journal of the International AIDS Society. 2019;22(10):e25389.
20. Rusie LK, Orengo C, Burrell D, Ramachandran A, Houlberg M, Keglovitz K, et al. Preexposure Prophylaxis Initiation and Retention in Care Over 5 Years, 2012-2017: Are Quarterly Visits Too Much? Clin Infect Dis. 2018;67(2):283-7.
21. Rotsaert A, Reyniers T, Jacobs BKM, Vanbaelen T, Burm C, Kenyon C, et al. PrEP user profiles, dynamics of PrEP use and follow-up: a cohort analysis at a Belgian HIV centre (2017-2020). Journal of the International AIDS Society. 2022;25(7):e25953.
22. Grinsztejn B, Hoagland B, Moreira RI, Kallas EG, Madruga JV, Goulart S, et al. Retention, engagement, and adherence to pre-exposure prophylaxis for men who have sex with men and transgender women in PrEP Brasil: 48 week results of a demonstration study. Lancet HIV. 2018;5(3):e136-e45.
23. Hosek SG, Rudy B, Landovitz R, Kapogiannis B, Siberry G, Rutledge B, et al. An HIV Preexposure Prophylaxis Demonstration Project and Safety Study for Young MSM. J Acquir Immune Defic Syndr. 2017;74(1):21-9.
24. Kwan TH, Lui GCY, Lam TTN, Lee KCK, Wong NS, Chan DPC, et al. Comparison between daily and on-demand PrEP (pre-exposure prophylaxis) regimen in covering condomless anal intercourse for men who have sex with men in Hong Kong: A randomized, controlled, open-label, crossover trial. Journal of the International AIDS Society. 2021;24(9):e25795.
25. Traikiatphum J, Wongharn P, Moonwong J, Songtaweesin WN, Vitsupakorn S, Premgamone A, et al. Retention in event-driven PrEP among young Thai men who have sex with men at risk of HIV acquisition. International journal of STD & AIDS. 2022;33(8):799-805.
26. Centers for Disease Control and Prevention. US Public Health Service: Preexposure prophylaxis for the prevention of HIV infection in the United States—2017 Update: a clinical practice guideline. Available from: https://www.cdc.gov/hiv/pdf/risk/prep/cdc-hiv-prep-guidelines-2017.pdf.
27. Chan PA, Patel RR, Mena L, Marshall BD, Rose J, Sutten Coats C, et al. Long-term retention in pre-exposure prophylaxis care among men who have sex with men and transgender women in the United States. Journal of the International AIDS Society. 2019;22(8):e25385.
28. General Statistics Office of Vietnam - Ministry of Planning and Investment. Announcement of population survey of life costing Hanoi2022 [Available from: https://www.gso.gov.vn/du-lieu-va-so-lieu-thong-ke/2022/06/thong-cao-bao-chi-ket-qua-khao-sat-muc-song-dan-cu-2021/.
29. The World Bank Group. Official exchange rate (LCU per US$, period average) 2022 [cited 2022 10 September]. Available from: https://data.worldbank.org/indicator/PA.NUS.FCRF.
30. World Bank. GDP per capita (current US$) 2023 [cited 2022 16 May]. Available from: https://data.worldbank.org/indicator/NY.GDP.PCAP.CD.

For peer review only - http://bmjopen.bmj.com/site/about/guidelines.xhtml

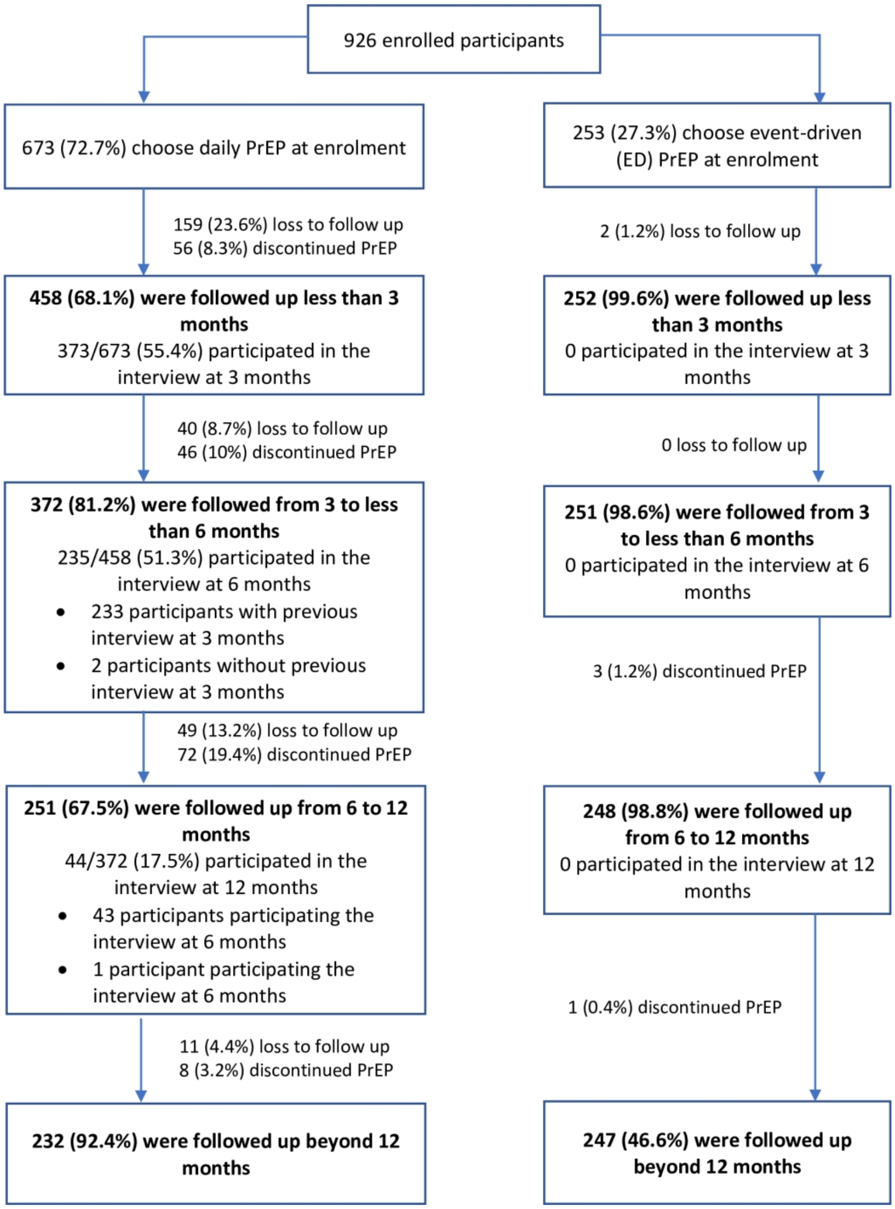

Figure 1. Flowchart of study participants.

159x214mm (300 x 300 DPI)

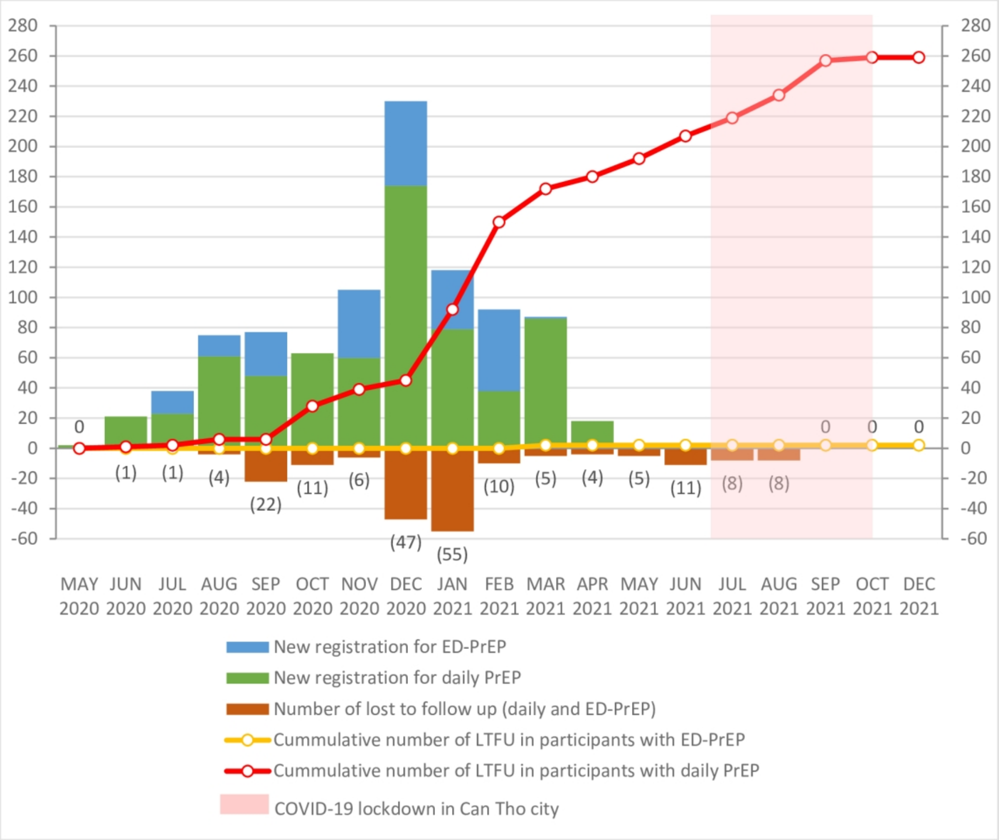

Figure 2. Number of new PrEP initiation and lost to follow up after the study enrolment

159x134mm (300 x 300 DPI)

Supplementary table 1. Baseline characteristic of participants using daily PrEP by the retention (excluding participants who decide to discontinue PrEP due to decreased HIV risk perception)

	All participants (n=491)	Retained in care (n=232)	Lost to follow up (n=259)	P value
**Sex assigned at birth**				
Male	465 (94.7%)	220 (94.8%)	245 (94.6%)	0.908
Female	26 (5.3%)	12 (5.2%)	14 (5.4%)	
**Sexual partners**				
No answer	1 (0.2%)	1 (0.4%)	0 (0.0%)	0.295
Men exclusively	419 (85.3%)	202 (87.1%)	217 (83.8%)	
Men and women	71 (14.5%)	29 (12.5%)	42 (16.2%)	
**HIV exposure within the past 3 days**				
No HIV exposure	488 (99.4%)	231 (99.6%)	257 (99.2%)	0.637
HIV exposure	2 (0.4%)	1 (0.4%)	1 (0.4%)	
No answer	1 (0.2%)	0 (0.0%)	1 (0.4%)	
**Frequency of sexual activity**				
≤ 2 times per week	169 (34.4%)	107 (46.1%)	62 (23.9%)	<0.001
>2 times per week	264 (53.8%)	105 (45.3%)	159 (61.4%)	
No answer	58 (11.8%)	20 (8.6%)	38 (14.7%)	
**Having sex without condom with people who were at risk of HIV within the past 6 months**				
No	130 (26.5%)	53 (22.8%)	77 (29.7%)	0.060
Yes	312 (63.5%)	160 (69.0%)	152 (58.7%)	
No answer	49 (10.0%)	19 (8.2%)	30 (11.6%)	
**Number of sexual partner within the past 6 months**				
1 sexual partner	115 (23.4%)	64 (27.6%)	51 (19.7%)	0.102
At least 2 sexual partners	370 (75.4%)	166 (71.6%)	204 (78.8%)	
No answer	6 (1.2%)	2 (0.9%)	4 (1.5%)	
**Having sex for money or gifts within the past 6 months**				
No	461 (93.9%)	225 (97.0%)	236 (91.1%)	0.017
Yes	21 (4.3%)	6 (2.6%)	15 (5.8%)	
No answer	9 (1.8%)	1 (0.4%)	8 (3.1%)	
**Diagnosis and/or treatment with an STI within the past 6 months**				
No	439 (89.4%)	208 (89.7%)	231 (89.2%)	0.006
Yes	39 (7.9%)	23 (9.9%)	16 (6.2%)	
No answer	13 (2.6%)	1 (0.4%)	12 (4.6%)	
**Sharing needles with other people within the past 6 months**				
No	486 (99.8%)	230 (100.0%)	256 (99.6%)	0.344

For peer review only

Yes	0 (0.0%)	0 (0.0%)	0 (0.0%)	
No answer	1 (0.2%)	0 (0.0%)	1 (0.4%)	
**Used PrEP within the past 6 months**				
No	451 (92.6%)	199 (86.5%)	252 (98.1%)	<0.0001
Yes	33 (6.8%)	31 (13.5%)	2 (0.8%)	
No answer	3 (0.6%)	0 (0.0%)	3 (1.2%)	
**Willingness to pay for PrEP**				
< $4.25/month	100 (20.4%)	44 (19.0%)	56 (21.6%)	0.101
$4.25-$14.89/month	302 (61.5%)	138 (59.5%)	164 (63.3%)	
$14.89-42.55$/month	67 (13.6%)	41 (17.7%)	26 (10.0%)	
>$42.55/month	1 (0.2%)	1 (0.4%)	0 (0.0%)	
Don’t want/unable to pay for PrEP	21 (4.3%)	8 (3.4%)	13 (5.0%)	

For peer review only

Supplementary table 3. Concerns about potential barriers to access PrEP at baseline

	All	Daily PrEP	ED-PrEP
Anticipated barrier to PrEP	338 (36.5%)	265 (39.4%)	73 (28.9%)
Concern about side effects	9 (1.0%)	9 (1.3%)	0 (0.0%)
Difficult in managing the time for visiting clinic and drug pick up PrEP	8 (0.9%)	5 (0.7%)	3 (1.2%)
Concerned about stigma/community perception	8 (0.9%)	8 (1.2%)	0 (0.0%)
Concerned about family finding out	6 (0.6%)	6 (0.9%)	0 (0.0%)
Concern about cost	6 (0.6%)	6 (0.9%)	0 (0.0%)
Concerned about stigma/community perception	4 (0.4%)	4 (0.6%)	0 (0.0%)
Difficulty in planning sex in advance	3 (0.3%)	1 (0.1%)	2 (0.8%)
Concerned about carrying the pills	2 (0.2%)	2 (0.3%)	0 (0.0%)
Difficulty in taking the pills 2 hours before sex	1 (0.1%)	1 (0.1%)	0 (0.0%)
Difficulty in remembering to take tablet everyday	1 (0.1%)	0 (0.0%)	1 (0.4%)
Drug storage	1 (0.1%)	1 (0.1%)	0 (0.0%)
Difficulty in managing the time for visiting clinic and drug pick up	1 (0.1%)	0 (0.0%)	1 (0.4%)
Concerned on the comorbidity of asthma	1 (0.1%)	0 (0.0%)	1 (0.4%)
Difficulty in transportation of PrEP	0 (0.0%)	0 (0.0%)	0 (0.0%)
Concerned about partner finding out	0 (0.0%)	0 (0.0%)	0 (0.0%)
Difficulty in remembering to take the following doses	0 (0.0%)	0 (0.0%)	0 (0.0%)
Difficulty in accessing STI, hepatitis testing and/or treatment	0 (0.0%)	0 (0.0%)	0 (0.0%)

Supplementary table 3. Challenges reported by daily PrEP users at 3, 6 and 12 months interviews

	3 months N= 373	6 months N=235	12 months N=44
**Willing to pay for PrEP per month**			

For peer review only

Don't want/unable to pay for PrEP	19 (5.1%)	9 (3.8%)	0 (0.0%)
>$42.55	1 (0.3%)	1 (0.4%)	0 (0.0%)
$14.89-42.55$	45 (12.1%)	36 (15.3%)	21 (47.7%)
$4.25-$14.89	240 (64.3%)	152 (64.7%)	22 (50.0%)
< $4.25	68 (18.2%)	36 (15.3%)	1 (2.3%)
**In the past 3 months, do you use daily PrEP or ED PrEP?**			
Both daily and ED PrEP	17 (4.6%)	3 (1.3%)	0 (0.0%)
Daily PrEP	296 (79.4%)	190 (80.9%)	36 (81.8%)
Any difficulties in taking PrEP medication in the past 3 months	110 (29.5%)	22 (9.4%)	13 (29.5%)
Forgot to take one or more PrEP doses	37 (9.9%)	9 (3.8%)	5 (11.4%)
Fitting PrEP into daily routine (for daily PrEP users)	7 (1.9%)	1 (0.4%)	1 (2.3%)
Unsure when to take PrEP medications	1 (0.3%)	1 (0.4%)	0 (0.0%)
Didn't know what to do about a missed dose	5 (1.3%)	3 (1.3%)	2 (4.5%)
Concerns about interactions with other medications	11 (2.9%)	5 (2.1%)	7 (15.9%)
Concerns about interactions with alcohol	2 (0.5%)	3 (1.3%)	2 (4.5%)
Concerns about interactions with hormonal therapy	1 (0.3%)	1 (0.4%)	0 (0.0%)
Have you had any difficulties accessing PrEP services?	131 (35.1%)	53 (22.6%)	18 (40.9%)
Difficult in managing the time for visiting clinic and drug pick up	37 (9.9%)	18 (7.7%)	6 (13.6%)
Transportation difficulty	11 (2.9%)	13 (5.5%)	13 (29.5%)
Concerned about partner finding out	5 (1.3%)	5 (2.1%)	1 (2.3%)
Concerned about family finding out	32 (8.6%)	12 (5.1%)	2 (4.5%)
Concerned about stigma/community perception	9 (2.4%)	5 (2.1%)	0 (0.0%)
Difficulty in remembering to take tablet everyday	17 (4.6%)	2 (0.9%)	0 (0.0%)
Carrying the pills with me	13 (3.5%)	7 (3.0%)	5 (11.4%)
Concern about side effects	52 (13.9%)	12 (5.1%)	4 (9.1%)
Concern about cost	9 (2.4%)	1 (0.4%)	0 (0.0%)
Difficulty in accessing STI, Hepatitis testing and/or treatment	5 (1.3%)	3 (1.3%)	1 (2.3%)
Health services closed due to Covid-19	33 (8.8%)	21 (8.9%)	16 (36.4%)

For peer review only

Not able to leave home due to Covid-19 lockdown	34 (9.1%)	27 (11.5%)	15 (34.1%)
**Side effects**			
Sleepy	1 (0.3%)	0 (0.0%)	0 (0.0%)
Nausea	12 (3.2%)	1 (0.4%)	1 (2.3%)
Muscle pain	1 (0.3%)	0 (0.0%)	0 (0.0%)
Loss of appetite	2 (0.5%)	0 (0.0%)	0 (0.0%)
Headache	3 (0.8%)	3 (1.3%)	2 (4.5%)
Dry lips	5 (1.3%)	2 (0.9%)	1 (2.3%)
Burning	5 (1.3%)	2 (0.9%)	4 (9.1%)
Acne	0 (0.0%)	0 (0.0%)	2 (4.5%)
Hyperpigmentation	0 (0.0%)	0 (0.0%)	0 (0.0%)
Fatigue	7 (1.9%)	6 (2.5%)	1 (2.3%)
Dizziness	7 (1.9%)	0 (0.0%)	0 (0.0%)
Dry skin	0 (0.0%)	0 (0.0%)	1 (2.3%)

Supplementary table 4. Number of participants switching between daily and ED-PrEP during the following up period

	**Regimen**	**Participants preferred a daily PrEP regimen**	
**During the first 3 months of PrEP use**	Daily PrEP	296	80.0%
	ED-PrEP	57	15.4%
	Switching between daily and ED-PrEP	17	4.6%
	Subtotal	370	100.0%
**Between 3 months and 6 months of PrEP use**	Daily PrEP	190	81.2%
	ED-PrEP	41	17.5%

For peer review only

	Switching between daily and ED-PrEP	3	1.3%
	Subtotal	234	100.0%
**Between 9 and 12 months of PrEP use**	Daily PrEP	36	83.7%
	ED-PrEP	7	16.3%
	Switching between daily and ED-PrEP	0	0.0%
	Subtotal	43	100.0%

For peer review only

STROBE Statement—checklist of items that should be included in reports of observational studies

	Item No	Recommendation	Page No
**Title and abstract**	1	(*a*) Indicate the study's design with a commonly used term in the title or the abstract	1
		(*b*) Provide in the abstract an informative and balanced summary of what was done and what was found	2
**Introduction**			
Background/rationale	2	Explain the scientific background and rationale for the investigation being reported	4
Objectives	3	State specific objectives, including any prespecified hypotheses	5
**Methods**			
Study design	4	Present key elements of study design early in the paper	5
Setting	5	Describe the setting, locations, and relevant dates, including periods of recruitment, exposure, follow-up, and data collection	5
Participants	6	(*a*) Give the eligibility criteria, and the sources and methods of selection of participants	5
Variables	7	Clearly define all outcomes, exposures, predictors, potential confounders, and effect modifiers. Give diagnostic criteria, if applicable	5
Data sources/ measurement	8*	For each variable of interest, give sources of data and details of methods of assessment (measurement). Describe comparability of assessment methods if there is more than one group	*6*
Bias	9	Describe any efforts to address potential sources of bias	9
Study size	10	Explain how the study size was arrived at	5
Quantitative variables	11	Explain how quantitative variables were handled in the analyses. If applicable, describe which groupings were chosen and why	NA
Statistical methods	12	(*a*) Describe all statistical methods, including those used to control for confounding	6
		(*b*) Describe any methods used to examine subgroups and interactions	6
		(*c*) Explain how missing data were addressed	Fig 11
		(*d*) If applicable, describe analytical methods taking account of sampling strategy	NA
		(*e*) Describe any sensitivity analyses	NA
**Results**			
Participants	13*	(a) Report numbers of individuals at each stage of study—eg numbers potentially eligible, examined for eligibility, confirmed eligible, included in the study, completing follow-up, and analysed	6
		(b) Give reasons for non-participation at each stage	6
		(c) Consider use of a flow diagram	
Descriptive data	14*	(a) Give characteristics of study participants (eg demographic, clinical, social) and information on exposures and potential confounders	6
		(b) Indicate number of participants with missing data for each variable of interest	6
Outcome data	15*	Report numbers of outcome events or summary measures	6

For peer review only

Main results	16	(*a*) Give unadjusted estimates and, if applicable, confounder-adjusted estimates and their precision (eg, 95% confidence interval). Make clear which confounders were adjusted for and why they were included	7
		(*b*) Report category boundaries when continuous variables were categorized	NA
		(*c*) If relevant, consider translating estimates of relative risk into absolute risk for a meaningful time period	NA
Other analyses	17	Report other analyses done—eg analyses of subgroups and interactions, and sensitivity analyses	7
**Discussion**			
Key results	18	Summarise key results with reference to study objectives	8
Limitations	19	Discuss limitations of the study, taking into account sources of potential bias or imprecision. Discuss both direction and magnitude of any potential bias	9
Interpretation	20	Give a cautious overall interpretation of results considering objectives, limitations, multiplicity of analyses, results from similar studies, and other relevant evidence	9
Generalisability	21	Discuss the generalisability (external validity) of the study results	9
**Other information**			
Funding	22	Give the source of funding and the role of the funders for the present study and, if applicable, for the original study on which the present article is based	8

*Give information separately for exposed and unexposed groups.

Note: An Explanation and Elaboration article discusses each checklist item and gives methodological background and published examples of transparent reporting. The STROBE checklist is best used in conjunction with this article (freely available on the Web sites of PLoS Medicine at http://www.plosmedicine.org/, Annals of Internal Medicine at http://www.annals.org/, and Epidemiology at http://www.epidem.com/). Information on the STROBE Initiative is available at www.strobe-statement.org.

For peer review only - http://bmjopen.bmj.com/site/about/guidelines.xhtml
